# Can Niche Modeling and Geometric Morphometrics Document Competitive Exclusion in a Pair of Subterranean Rodents (Genus *Ctenomys*) with Tiny Parapatric Distributions?

**DOI:** 10.1038/s41598-017-16243-2

**Published:** 2017-11-24

**Authors:** Bruno B. Kubiak, Eliécer E. Gutiérrez, Daniel Galiano, Renan Maestri, Thales R. O. de Freitas

**Affiliations:** 1 Programa de Pós-Graduação em Biologia Animal, Departamento de Zoologia Universidade Federal do Rio Grande do Sul, Av. Bento Gonçalves 9500, 91501-970 Porto Alegre, Brazil; 2 Departamento de Ciências Biológicas da Universidade Regional Integrada do Alto Uruguai e das Missões – Campus de Frederico Westphalen, Av. Assis Brasil 709, 98400-000 Frederico Westphalen, Brazil; 3 Programa de Pós-Graduação em Biodiversidade Animal, Centro de Ciências Naturais e Exatas, Av. Roraima n. 1000, Universidade Federal de Santa Maria, Santa Maria, RS 97105-900 Brazil; 4 Programa Nacional de Pós Doutorado em Ecologia, Departamento de Zoologia, Instituto de Ciências Biológicas, Campus UnB, Universidade de Brasília, Asa Norte 70910-900 Brasília, DF Brazil; 5 Division of Mammals, Department of Vertebrate Zoology, National Museum of Natural History, Smithsonian Institution, Washington, DC USA; 6 Programa de Pós-Graduação em Ciências Ambientais – Universidade Comunitária da Região de Chapecó, - Avenida Senador Attílio Fontana, 591-E, CEP 89809-000 Chapecó, SC Brazil; 7 Programa de Pós-Graduação em Genética e Biologia Molecular, Departamento de Genética, Universidade Federal do Rio Grande do Sul – Av. Bento Gonçalves, 9500, CEP 91501-970 Porto Alegre, RS Brazil

## Abstract

Species with similar ecological requirements coexisting in the same geographic region are prone to competitively exclude each other. Alternatively, they may coexist if character displacement acts to change the niche requirements of one or both species. We used two methodological approaches (ecological niche modeling [ENM] and geometric morphometrics) to test two hypotheses: given their behavioral, morphological, and ecological similarities, one species competitively excludes the other; and, character displacement enables their coexistence at two sites in which the species are known to occur in sympatry. The results from the ENM-based approach did not provide evidence for competitive exclusion; however, the morphometric analyses documented displacement in size of *C*. *minutus*. This result, suggests that *C*. *minutus* might exclude *C*. *flamarioni* from areas with softer soils and higher food availability. We stress the importance of using multiple methodological approaches when testing prediction of competitive exclusion. However, both methods had limited explanatory power given that the focal species possess truly peculiar distributions, being largely parapatric and restricted to narrow, small geographic areas with a strange distribution and there is a need to search for additional methods. We discuss the idiosyncrasy of the ENM-based approach when applied to organisms with subterranean habits.

## Introduction

Species’ distributions are influenced by history, climate biotic interactions, and other factors^1^. Among the most studied biotic interactions that affect species’ distributions is competition, being competitive exclusion its most extreme manifestation, where a superior competitor excludes another species from a geographic area. According to the principle of competitive exclusion, species that exhibit highly similar ecological requirements cannot coexist when resources are limited^2,3^. The geographic ranges of closely related, morphologically similar species pairs have interested ecologists and evolutionary biologists since long ago. When those ranges broadly overlap, the species often use different habitats or differ in behavior, which lessens the strength of competition^4–7^. These behavioral differences may be followed by morphological differentiation^8–10^, a phenomenon known as character displacement (see below).

Significant advances in ecological niche modeling (ENM) have been achieved in the 21^st^ century, and the development of approaches to study biotic interactions has not been an exception. These efforts have yielded examples of biotic interactions affecting species distribution. Indirect examples of this effect have been provided by studies that showed that incorporating proxies of biotic interactions as predictor variables can enhance model predictive ability^11–15^. Others, more direct, examples have come from studies that employed ENM to unveil evidences of factual, or possible, species interactions^7,16–18^.

A method for testing the geographic predictions of competitive exclusion and release has been developed based on analyses of both geographic projection of ENMs and occurrence records of a pair of potentially competing species^16,18^. These tests are applicable when the following geographic requirements are met^16,19^: (1) the occupied distributional areas (G_O_; see Peterson *et al*.^19^, p. 30) of the two species do not broadly overlap; however, (2) their abiotically suitable areas (G_A_) overlap, forming what has been termed as ‘areas of potential sympatry’^16^; (3) within the areas of potential sympatry, contact zones exist, where competition could take place (this allows for testing the geographic prediction of competitive exclusion); and, (4) also within the areas of potential sympatry, should exist zones where only one species is present, where competitive release could take place (this allows for testing the geographic prediction of competitive release, see below). Logically, in addition to these geographic requirements, a credible case for the focal species to potentially be competitors should exist, for example due to a high morphological similarity and a close phylogenetic relationship.

The geographic prediction for competitive exclusion is that the putative superior species is more common—i.e., more than expected by chance in terms of proportion of unique collection localities—than the other species (i.e., the putatively inferior competitor) in areas of potential sympatry along contact zones^16^. The geographic prediction of competitive release states that, in zones within the areas of potential sympatry, where the putative superior competitor is absent, the putative inferior competitor inhabits conditions similar to those from which it is excluded in the contact zones^16,19^. Recently, Gutiérrez *et al*.^18^ proposed an extension to these tests in which the strength of model predictions are employed to visualize if environmental suitability may drive the outcome of the putative competitive exclusion (if any). The idea is to assess whether each species outcompete the other wherever the environmental conditions are more suitable for it than for the other species, or, alternatively, if the putative superior competitor excludes the putative inferior competitor even from areas more strongly predicted suitable for the latter.

Even in cases in which testing these predictions yield results suggestive of competitive exclusion and release, these analyses based on correlative modeling cannot demonstrate these phenomena. Nevertheless, this method can provide directional hypotheses that can then be tested via experimental field and laboratory studies^16,20–22^. However, when ENM-based analyses do not yield results congruent with the prediction for competitive exclusion, alternative hypotheses are necessary to explain how the focal species maintain their parapatric distributions around contact zones. Because of the limitations of the ENM-based method, and because experimental studies to test for competitive exclusion often are not feasible, here we also conducted a test for morphological character displacement based on geometric morphometrics data. In addition, we used natural history information for the species, including spatial distribution^23–27^, diet composition^28^, and microhabitats requirements^29,30^. The combination of both techniques^31^, together with natural history information, can provide stronger, and likely complementary evidence of competitive interactions than the use of any of them alone.

Character displacement is an evolutionary phenomenon caused by intense competitive interactions^8,9,32^. Brown and Wilson^8^ were the first to use the term “character displacement”, while hypothesizing that the evolution of differences in morphology lead to a reduction in the overlap in resource use and interspecific competition. They suggested that when two species involved in an intense competitive interaction, the species tend to exhibit marked differences in morphology in areas of sympatry, whereas outside of these areas their differences are smaller or even absent. Since then, numerous studies have explored the role of competition in the displacement of morphological traits^10,32–37^.

Subterranean rodents possess suitable characteristics for the objectives of the present study. The underground environment presents relatively simple, stable conditions and provides protection from predators^38^. These aspects probably explain the morphological convergences observed among subterranean rodent species, such as cylindrical body and anatomical reductions (eyes, tail, ears, etc.^38,39^). Besides these similarities, subterranean rodent species also exhibit distributional patterns that are predominantly allopatric or peripatric, with only a few cases of sympatry. In these latter cases the implicated species exhibit differentiation in the selection of microhabitats, thus allowing their coexistence^29,40–45^. In addition, two species from southern Brazil exhibit a partition in food items when occur in sympatry^28^. These aspects suggest that interspecific competition might be a strong force shaping the distribution of these rodents and that when species co-occur they exhibit strategies to avoid competition.

We studied two species of subterranean rodents, the tuco-tucos, *Ctenomys flamarioni* Travi, 1981 and *Ctenomys minutus* Nehring, 1887, and tested two hypotheses: (1) given their behavioral, morphological, and ecological similarities (reviewed below), one species competitively excludes the other from areas with suitable environmental conditions for both species around their known contact zones; or, alternatively (2) character displacement enables their coexistence in these areas (i.e., competitive exclusion does not take place). Thus, we tested for the geographic predictions of competitive exclusion and release based on a ENM-based method^16,19^, and employed geometric morphometrics to test if the two focal species are more morphologically distinct in areas of sympatry than in regions where they occur in allopatry. We assessed whether the combined use of these techniques allow for a better understanding of competitive interactions than the use of either of them alone. The results from these analytical approaches were then integrated with available natural history information, informing on diet and microhabitat partition.

## Results

### Species’ models and areas of potential sympatry

Our analyses for selecting optimal Maxent’s settings to model species’ abiotically suitable areas identified Linear and Quadratic features as the best performing combination of feature classes for both species. For *C*. *flamarioni* the optimal value for the regularization multiplier was 1, whereas for *C*. *minutus* it was 1.5 (see results of model tuning analyses in ENMeval in Supplementary Information Tables S1 and S2).

**Figure 1:**
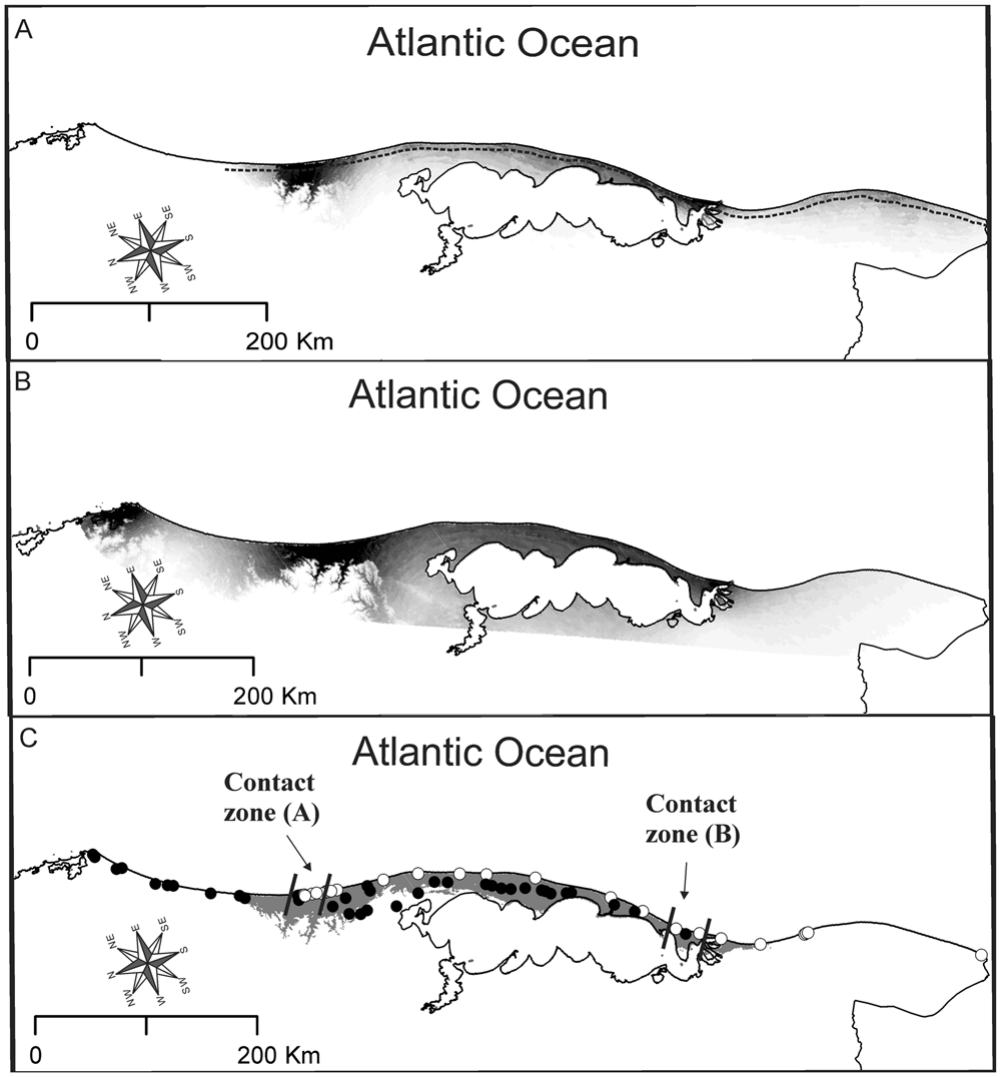
Ecological niche models projected (in binary format) on the coastal plains of southern Brazil: (**A**) final Maxent model of abiotically suitable areas for *Ctenomys flamarioni*; (**B**) final Maxent model of abiotically suitable areas for *C*. *minutus*, and (**C**) areas of potential sympatry for both species. Parallel lines indicate location of known contact zone in the north (**A**) and the south (**B**). Abiotically suitable areas are indicated with shades of gray; increasingly stronger predictions are indicated with pregressively darker shades. Areas of potential sympatry are those where suitable environmental conditions exist for both species. The dashed line in “A” indicates the approximate limits of the sand dunes. Withe circles represent localities of *C*. *flamarioni*; black circles reprensent localities of *C*. *minutus*. Maps were obtained from “© OpenStreetMap contributors” (available at: www.openstreetmap.org; Open Street Map is made available under the Open Database License: http://opendatacommons.org/licenses/odbl/1.0/. Any rights in individual contents of the database are licensed under the Database Contents License: http://opendatacommons.org/licenses/dbcl/1.0/; and http://mapas.mma.gov.br/i3geo/datadownload.htm), and edited with QGis 2.18 software. The images were also edited using Corel Draw graphics Suite (X5).

Final models of both species identified abiotically suitable areas in the coastal plain region of the southern extreme of Brazil. The final model of *C*. *minutus* predicted extensive areas as suitable along the coastal plain region, with strong predictions in sandy fields and in sandy dunes. On the other hand, the abiotically suitable areas identified by the final model of *C*. *flamarioni* almost exclusively included the sand dunes, where its predictions were stronger than those from the model of *C*. *minimus*. Only in two regions, located near the known contact zones, *C*. *flamarioni* had strong predictions in locations away from the sandy dunes (Fig. 1).

**Figure 2:**
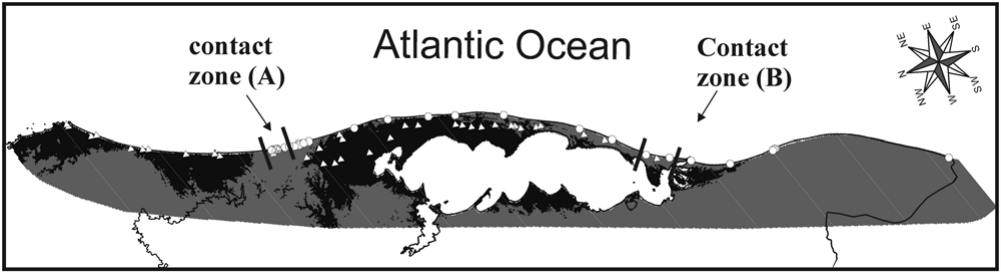
Binary representation of the climatic suitability for *Ctenomys flamarioni* and *C*. *minutus* in areas of potential sympatry. Circles represent records of *C*. *flamarioni*; triangles represent records of *C*. *minutus*. Sites (image pixels) with higher suitability values for *C*. *flamarioni* are indicated in grey; sites (image pixels) with higher suitability values for *C*. *minutus* are indicated with black. Maps were obtained from “© OpenStreetMap contributors” (available at: www.openstreetmap.org; Open Street Map is made available under the Open Database License: http://opendatacommons.org/licenses/odbl/1.0/. Any rights in individual contents of the database are licensed under the Database Contents License: http://opendatacommons.org/licenses/dbcl/1.0/; and http://mapas.mma.gov.br/i3geo/datadownload.htm), and edited with QGis 2.18 software. The images were also edited using Corel Draw graphics Suite (X5).

Once overlapped, binary projections of final models showed an extensive region of potential sympatry in the coastal plain of the Rio Grande do Sul state (Fig. 2). The northern boundary of this region is located near the state border between Rio Grande do Sul and Santa Catarina states, whereas the southern boundary is located near the shore of the Patos Lagoon. The environment in this region of potential sympatry is predominantly composed of sandy fields and sandy dunes.

### Test for competitive exclusion and release

**Figure 3:**
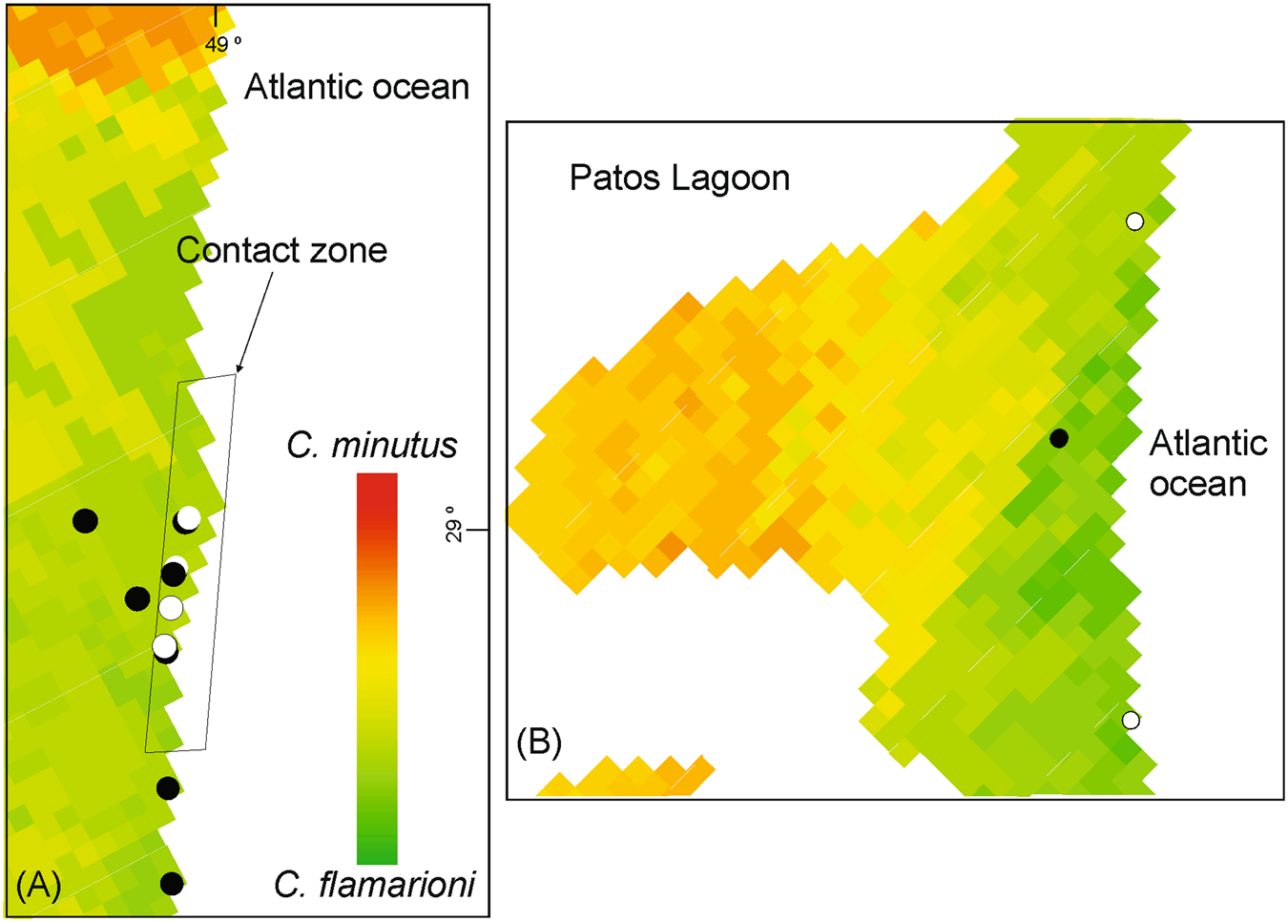
Comparison strenghts of of predicted environmental suitability for *C*. *flamarioni* and *C*. *minutus*. These comparisons are based on final models projected at the known conctact zones of the focal species. (**A**) shows models projected at the northern area, whereas (**B**) does so for the southern area. Withe circles reprensent localities of *C*. *flamarioni* and black circles represent localities of *C*. *minutus*. The rectangle in the figure (**A**) represents the limits of the contact zone between the species. Maps were obtained from “© OpenStreetMap contributors” (available at: www.openstreetmap.org; Open Street Map is made available under the Open Database License: http://opendatacommons.org/licenses/odbl/1.0/. Any rights in individual contents of the database are licensed under the Database Contents License: http://opendatacommons.org/licenses/dbcl/1.0/; and http://mapas.mma.gov.br/i3geo/datadownload.htm), and edited with QGis 2.18 software. The images were also edited using Corel Draw graphics Suite (X5).

A total of 61 localities of the focal species were present in areas of potential sympatry. *Ctenomys flamarioni* had a total of 24 localities in areas of potential sympatry, 18 of them away from the contact zones, 4 localities in the northern contact zone, and 2 localities in the southern contact zone. *Ctenomys minutus* had a total of 37 localities in areas of potential sympatry, 33 of them away from contact zones, 3 in the northern contact zone, and 1 locality in the southern contact zone (Fig. 3). The expected values for the exact binomial tests for *C*. *flamarioni* and *C*. *minutus* in the northern contact zone were 2.471 and 4.529, respectively, whereas in the southern contact zone they were 1.059 and 1.941, respectively. Neither in the northern (*P* = 0.2522) nor in the southern (*P* = 0.2858) contact zones the observed numbers of localities significantly deviated from expectations by chance.

Records of either species occurred in areas more strongly predicted suitable for that species, but not always. In areas of potential sympatry out of the contact zones, we found a few sites in which a species occurred despite climatic conditions were predicted more suitable for the other species (Fig. 1). That is, in a few sites *C*. *minutus* occurred despite conditions were more strongly predicted suitable for *C*. *flamarioni*, and, likewise, in a few sites *C*. *flamarioni* occurred despite conditions were more strongly predicted suitable for *C*. *minutus*. By contrast, in contact zones both species occurred in sites always more strongly predicted suitable for *C*. *flamarioni* (Figs 2 and 3).

### Character displacement

**Figure 4:**
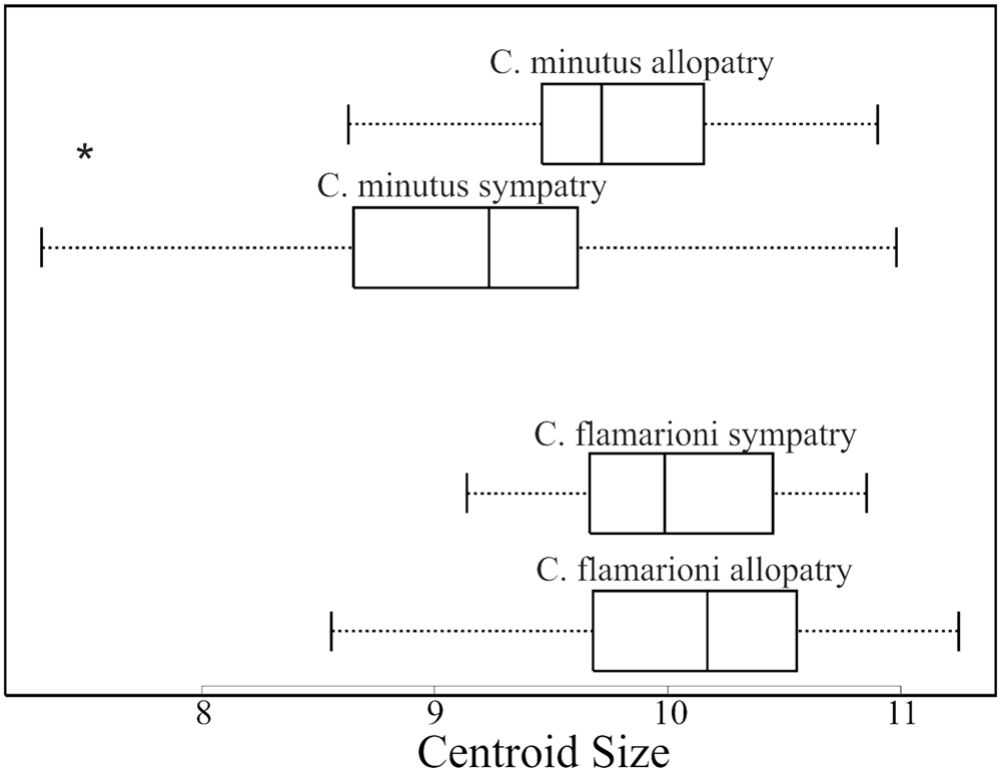
Boxplot showing skull centroid size variation in *Ctenomys flamarion* and *C*. *minutus* in sympatry and allopatry. Asterisk indicates a significant difference between groups. The central line show the median, and the square limits are showing the first and thrid quartiles, respectively.

**Figure 5:**
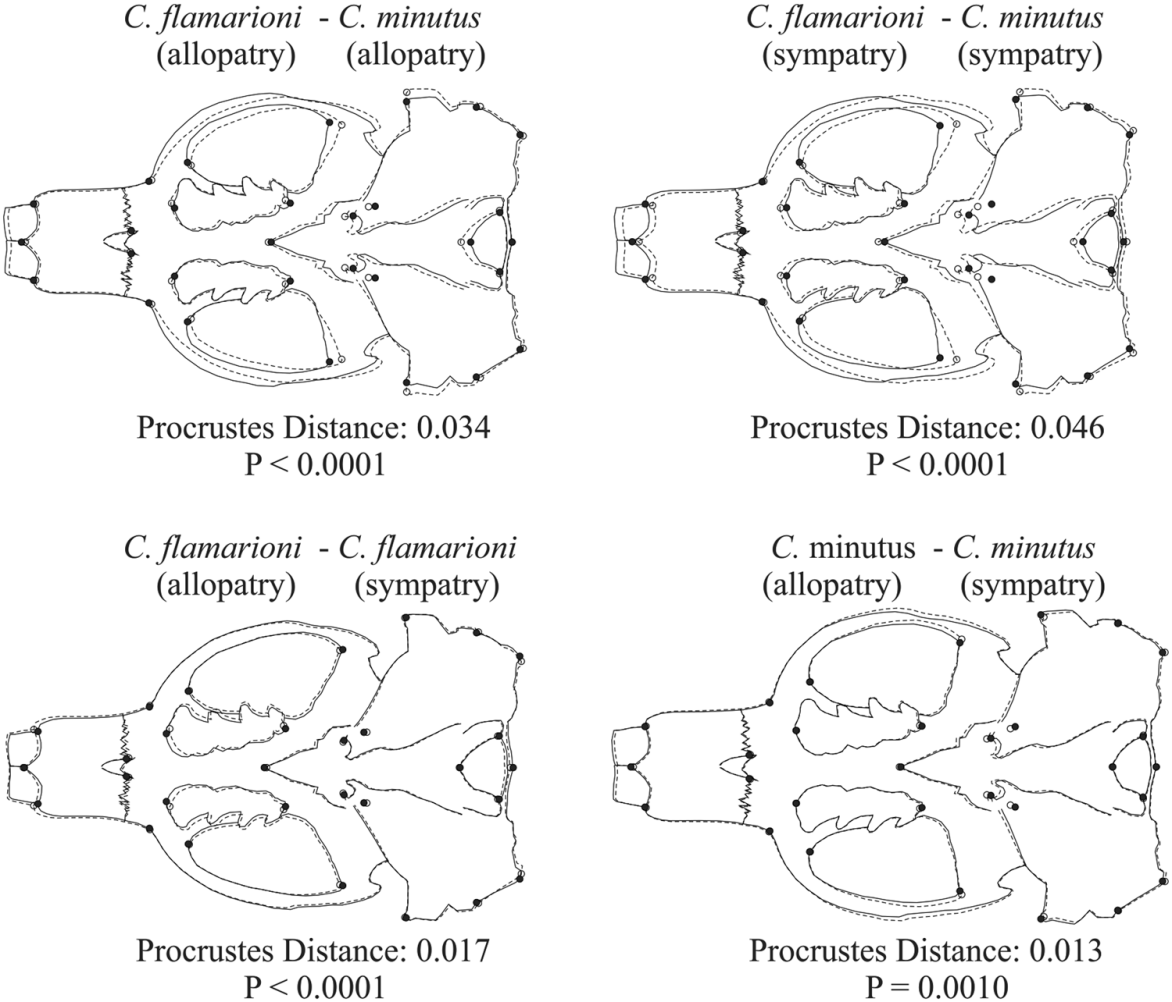
Procrustes distances between groups. The dotted line represents the first group and the continuous line represents the second. For example, in the first image the dotted line represents *Ctenomys flamarioni* in allopatry, whereas the continuous line represents *C*. *minutus* in allopatry.

We found significant differences in size between groups (Centroid size: F_3,81_ = 6.99, *P* = 0.0035; Skull length: F_3,81_ = 9.48, *P* ≤ 0.0001; Body mass: F_3,60_ = 4,61; *P* = 0.0057). Specimens of *C*. *minutus* from areas of actual sympatry with *C*. *flamarioni* have a smaller size than specimens from areas in which the species is in allopatry (Centroid size: 9.18 ± 0.85 and 9.74 ± 0.61, respectively, *P* = 0.036; Skull length: 471.23 ± 42.02 and 501.96 ± 32.54 mm, respectively, *P* = 0.029; Body mass: 186.82 ± 58.46 and 243.4 ± 55.99 g, respectively; *P* = 0.045). By contrast, size differences were not detected between specimens of *C*. *flamarioni* from sympatry or allopatry (Centroid size: 10.00 ± 0.53 and 10.30 ± 0.67, respectively, *P* = 0.999; Skull length: 521.29 ± 29.16 and 521 ± 34.33 mm, respectively, *P* = 1; Body mass: 240.15 ± 59.45 g and 241.21 ± 49.55 g, respectively; *P* = 0.74) (Fig. 4). All pairwise comparisons for shape differences yielded significant results (Fig. 5). In both species, we found that specimens in conditions of sympatry showed significant differences in mean shape when compared with specimens in conditions of allopatry (*C*. *flamarioni*: Procrutes distance = 0.017, *P* < 0.0001; *C*. *minutus*: Procrutes distance = 0.013, *P* = 0.001). More importantly, comparisons between the focal species revealed that *C*. *flamarioni* and *C*. *minutus* are more different when they are sympatry than when they are in allopatry (Procrutes distances = 0.046, *P* < 0.0001; Procrutes distances = 0.034, *P* < 0.0001, respectively). The bootstrap test showed that this difference is significantly higher than expected by chance (*P* = 0.001).

**Figure 6:**
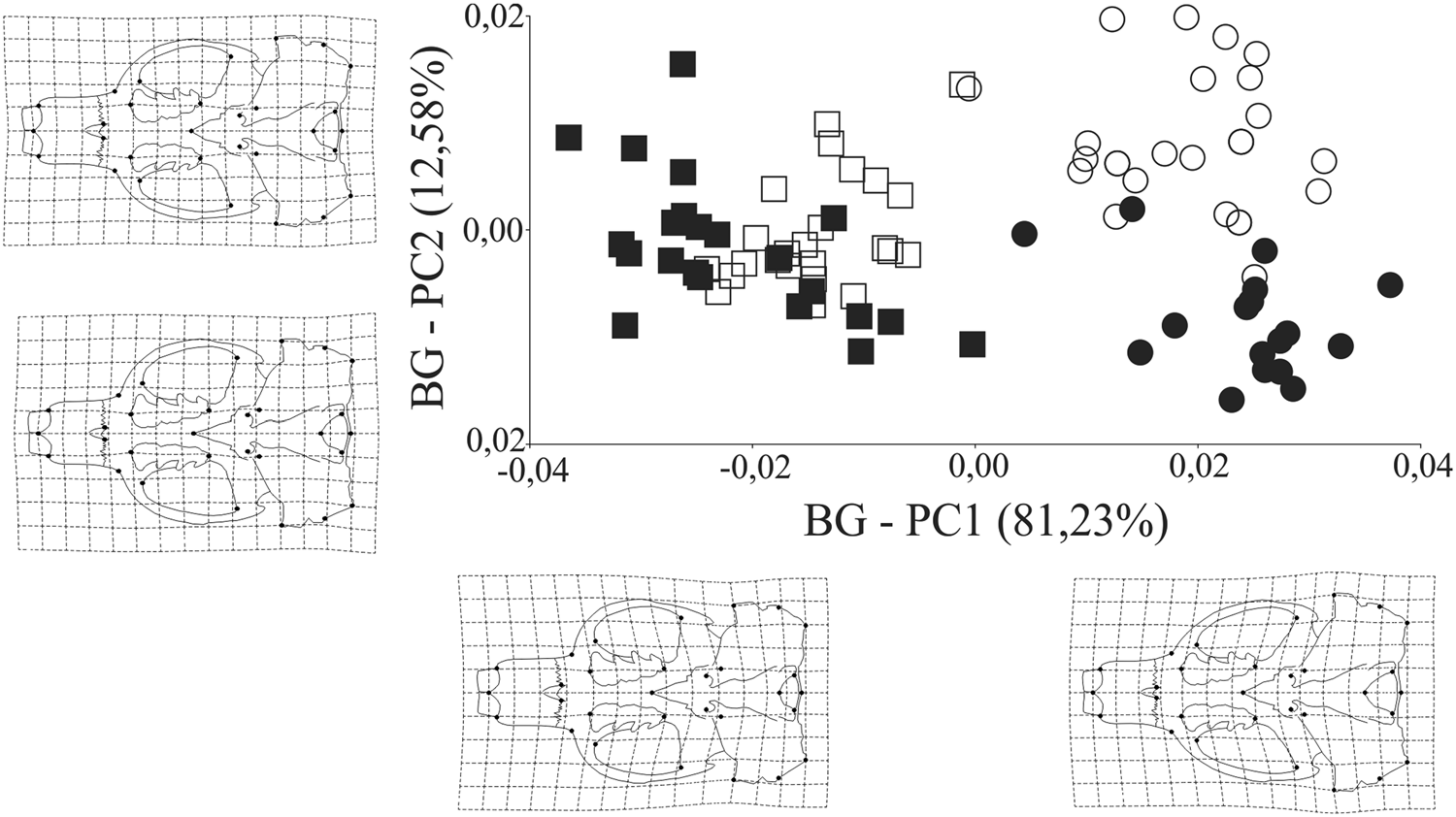
Scatter plot of the two first axes of a between-group principal component analysis for the ventral view of the skull for C. *flamarioni* and C. *minutus*. The predicted shape change along each axis is given. Solid circles represent *C*. *flamarioni* in allopatry; open circles represent *C*. *flamarioni* in sympatry; solid squares represent *C*. *minutus* in allopatry; open squares represent *C*. *minutus* in sympatry.

The BG-PCA showed variation between and within species in sympatry and allopatry (Fig. 6). The BG-PC1 explains 81.23% of the total variation. In this axis, differences between the two species are evident, and it is also observed a partial segregation between specimens of *C*. *minutus* in allopatric and sympatric conditions with respect to each other. Specimens with higher values on BG-PC1 show a more robust posterior part of the skull and increased zygomatic archs (towards the rear), causing the skull to present a slight inclination towards the rear. Moreover, specimens that have lower values on this axis present skulls that are relatively narrower posteriorly and relatively elongated anteriorly. In the BG-PC2 it is evident a difference between specimens of *C*. *flamarioni* in allopatric and sympatric conditions. BG-PC2 explains 12.58% of the total variation. In general, shape differences are accentuated for both species in sympatry.

## Discussion

Our ENM-based analyses yielded results that did not match the geographic pattern expected under competitive exclusion. Besides, both *C*. *flamarioni* and *C*. *minutus* possess records at sites more strongly predicted suitability for the other species, and this is incongruent with the possibility that competitive exclusion (if it would take place) were driven by the climatic conditions. In species pairs with small distributions, it is likely that scarcity of occurrences in areas of potential sympatry away from the contact zones could prevent obtaining expected values that otherwise might allow for the binomial test to yield significant results. However, despite the small, narrow distributions of our focal species, *C*. *flamarioni* and *C*. *minutus* possess enough (18 and 24, respectively) occurrences in areas of potential sympatry away from contact zones; hence, we consider that lack of data is not likely to be a potential explanation for the statistically insignificant results obtained. On the other hand, it is possible that the temperature and precipitation variables employed in the ENM analyses do not differentially affect the geographic distributions of the focal species, and that other factors might play a major role in this respect. Given that these species occur in close geographic proximity in a topographically homogeneous region, it is likely that they do not differ substantially in their climatic niches—although, as shown earlier, some significant differences exist with respect to the climatic conditions in which the focal species occur (see Supplementary Fig. S1 and Table S3), which enabled us to address the possibility that climatic factors could be driving a plausible competitive interaction between them. It is important to clarify that the fact that these ENM-based analyses, conducted with a specific set of bioclimatic variables, did not find patterns congruent with competitive exclusion does not mean that competitive exclusion might not be taking place.

Rather than climate, microhabitat characteristics might explain the observed geographic patterns. A recent study found that *C*. *flamarioni* and *C*. *minutus* show spatial segregation according to microhabitat characteristics^29^. Based on cases of habitat segregation previously reported for other subterranean rodents (ctenomyids^42,43^ and bathyergids^44^), several authors have suggested that habitat specialization might explain instances of sympatry between species in areas with mosaics of different habitats. On this regard, Vassallo^46^ conducted a study in which the focal species differed in size, and found that the removal of the larger species did not trigger the immigration of individuals of the smaller species into the emptied area. Vassallo^46^ concluded that differences in habitat preferences are more important than interspecific competition. On the other hand, Kubiak *et al*.^29^ demonstrated that either in sympatry or in allopatry, *C*. *minutus* selected areas characterized by higher amounts of plant biomass and higher grass cover when compared with areas occupied by *C*. *flamarioni*. By contrast, *C*. *flamarioni* showed a distinction in habitat selection when occurring in allopatry and sympatry; in allopatry, the species selects areas with high grass cover and is distributed on less hard soils in comparison to individuals in sympatry with *C*. *minutus*. This suggests that the selection of habitat by *C*. *flamarioni* might be influenced by the presence of a congeneric (and perhaps superior) competitor. However, a study with *C*. *flamarioni* and *C*. *minutus* in sympatry and allopatry suggests that co-occurrence may not influence home range size in these species, perhaps due to modifications presented by species that facilitate coexistence (e.g., microhabitat segregation and dietary modifications)^47^. Future studies should gather data on and consider the plausible role of soil properties, vegetation types, and characteristics of burrow systems explaining the parapatric distributions of these species.

Results from our character displacement analyses documented that the coexistence with *C*. *flamarioni* is coincident with, and it is likely the causal factor for, a reduction in the size in *C*. *minutus* (centroid size, skull length and body mass). This finding is congruent with our hypothesis that *C*. *flamarioni* and *C*. *minutus*, when in sympatry, should present morphological changes that enable their coexistence. The fact that *C*. *minutus* presents a reduction in size when in contact with *C*. *flamarioni* could be seen as an ecological advantage that allows the former species to continue using its preferred microhabitats even despite the presence of a larger congener. Coincidently, *C*. *flamarioni* experiences few or no changes in size and shape regardless of the presence or absence of *C*. *minutus*. This result supports the idea that the body size is important in competitive interspecific interactions^48^, where a bigger body size (*C*. *flamarioni*) provides competitive advantage over a smaller size (*C*. *minutus*) (e.g.^35,49,50^). However, reduction in body size might benefit the species experiencing it as such morphological change might enable its permanence at sites with high-quality microhabitat characteristics despite the presence of a bigger competitor. Browers and Brown^51^ found that granivorous rodents in dessert areas of the southwestern of the US tend to compete intensely when they exhibit similar body sizes (below a 1.5 ratio). These authors considered that this intense competition make unlikely that these species occur in sympatry. Our focal species show a pattern in agreement with Browers and Brown’s consideration, as both are parapatric along most of their distributions. The coexistence of both species at the contact zones might be explained by two not mutually exclusive factors: (1) the focal species forage for different food when in sympatry (see Brown *et al*.^52^), with *C*. *minutus* replacing items in its diet, and *C*. *flamarioni* decreasing the number of consumed plant species^28^; (2) the focal species use different microhabitats, as previously discussed, in terms of soil hardness and plant biomass^29^.

When we analyze observations of microhabitat selection and morphology for the areas of sympatry, we found that in the sandy dunes both species have different responses: *C*. *flamarioni* selects different microhabitats while *C*. *minutus* does not^29^, and *C*. *minutus* present morphological modifications (i.e. reduction of centroid size, skull length and body mass), while *C*. *flamarioni* does not. This may be a result of temporal segregation of microhabitats. Displacement of morphological characters might reflect changes resulting from many generations of habitat segregation between *C*. *flamarioni* and *C*. *minutus* in the sympatry area. The plausible effects of both of these phenomena (i.e., spatial segregation and morphological differentiation) might point in the same direction, with the co-occurrence of the species causing an ecological shift in the known zones of sympatry.

Our results highlight the importance of using multiple analytical methodological approaches and natural history information when testing predictions of interspecific competition, and document limitations of ENM when dealing with organisms with particularly small distributions, subterranean habits, or both. The ENM-based approach did not yield results congruent with the geographic pattern expected under competitive exclusion; the use of this method alone would have fail to reveal the competitive interaction suggested by the results of the morphometric analyses. Nonetheless, even the character displacement in these species were not remarkable, instead, they demonstrate only small differences in size and skull shape, indicating either weak competitive interactions or a recent contact between the species. Whereas the ENM-based approach is able to detect geographic patterns consistent with competitive exclusion^16,18^, it might fail to do so in cases in which the climatic variables employed are not important—or are substantially less important than other factors—in determining the spatial segregation observed between the focal species. This type I error is expected to occur more frequently in analyses focused on species with highly similar climatic niches, typically with small distributional ranges in topographically homogeneous regions, like is in the case of *C*. *flamarioni* and *C*. *minutus*. Consequently, we encourage the use of additional approaches when studying competitive interactions, especially at low geographic scales.

The use of geometric morphometrics, as well as examination of literature on microhabitat use by the focal species, suggests that they experience competitive exclusion. Whereas *C*. *flamarioni* might be a superior competitor given its bigger size, *C*. *minutus* always access its preferred microhabitat even at sites of demonstrated sympatry through a reduction in its size. Both species demonstrate ability to occupy sandy dunes habitat, however, they are not able to co-occur along this habitat: between the two known contact zones, no records of *C*. *minutus* was found for over 20 years of studies in areas with sandy dunes^23,25–27,30,53^, where *C*. *flamarioni* is widely recorded^24,29,54^ (Thales de Freitas personal observations). In contrast, *C*. *minutus* ^23,25–27,30,53^ is broadly recorded in sandy dunes north of both known contact zones, while *C*. *flamarioni* ^24,29,54^ has no record in these places. Character displacement results suggest modifications in the size of *C*. *minutus* when in contact with *C*. *flamarioni*, however, do not allow us to affirm that the current distribution of species is a result of competition. This along with the fact that the ENM approach based on climatic variables-only might be unable to detect patterns congruent with competition, even if competitive exclusion is manifested in the study system, document the limitation of macroecological tools when applied to species with markedly reduced distributions. In these systems, it seems necessary to use environmental variables more directly associated with underground conditions, as well as direct fieldwork designed to observe behavioral characteristics of the species. Obtaining data on population densities, age structure, and characteristics of burrow systems throughout the range of the focal species could also be highly insightful to understand the role of competition shaping the distributions of the species.

## Materials and Methods

### Focal species and study region

**Figure 7:**
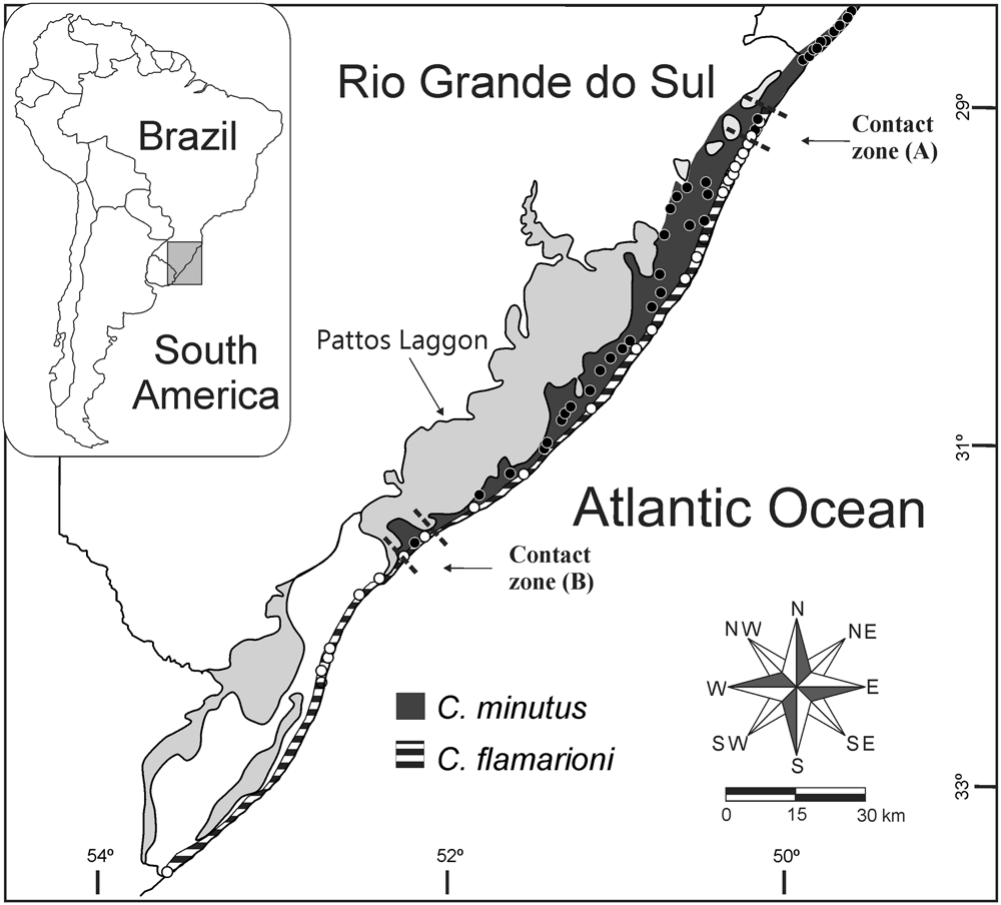
Localities of *Ctenomys flamarioni* (withe circles) and *Ctenomys minutus* (black circles) and study region used to calibrate models of the abiotically suitable areas. Areas in black and white show the realized distribution of *C*. *flamarioni*; areas in black represent the realized distribution of *C*. *minutus*; areas in gray indicate the location of ponds. Maps were obtained from “© OpenStreetMap contributors” (available at: www.openstreetmap.org; Open Street Map is made available under the Open Database License: http://opendatacommons.org/licenses/odbl/1.0/. Any rights in individual contents of the database are licensed under the Database Contents License: http://opendatacommons.org/licenses/dbcl/1.0/; and http://mapas.mma.gov.br/i3geo/datadownload.htm), and edited with QGis 2.18 software. The images were also edited using Corel Draw graphics Suite (X5).

Two species of subterranean rodents of the genus *Ctenomys* represent excellent candidates for testing the geographic predictions of competitive exclusion and displacement of morphological traits. The genus *Ctenomys*, commonly called tuco-tuco, with approximately 70 species, is widely distributed throughout South America^55^. These species are predominantly solitary, possess limited mobility and patchy distributions of local populations, and typically present allopatric distributions^39^. Only four species of *Ctenomys* are currently known to occur sympatrycaly with other congeners, *C*. *australis* Rusconi, 1934 with *C*. *talarum Thomas*, *1989*, and *C*. *flamarioni* with *C*. *minutus* ^29,42,43,56,57^. The latter two, *C*. *flamarioni* and *C*. *minutus*, our focal species, occur in the southern Brazilian coastal plain. This region is characterized by its geomorphology being constantly influenced by fluctuations of the Atlantic Ocean, which formed a mosaic of lakes and lagoons in two main environments: sandy dunes (beaches) and sandy fields^58^. Looking east to west, the landscape is formed by the Atlantic Ocean, followed by sandy dunes—beaches that can range from a few meters to about 200 meters width—and sandy fields (Fig. 7 and see Supplementary Information Fig. S2). The climate is mild mesothermal, wet without dry periods, and the vegetation consists of a mosaic of dune vegetation, sandy fields and “restinga” forest^59^, with a prevalence of herbaceous species over shrubs^60,61^. *Ctenomys flamarioni* is endemic to coastal sand-dune grasslands in the Rio Grande do Sul state, and its range, which extends for about 560 km, is bounded by the city of Arroio Teixeira on the north and by the Chuí River on the south^23,24^. *Ctenomys minutus* inhabits only the sand fields in the southern portion of its range, whereas in its northern portion the species inhabits the first-dune line, predominantly without presence of *Ctenomys flamarioni*. *Ctenomys minutus* occurs from Jaguaruna beach in the Santa Catarina state to the town of São José do Norte in the Rio Grande do Sul state, extending along more than 500 km^25–27^. These habitats greatly differ in their amount of plant biomass, with the sand fields having higher above and belowground plant biomass than the sand dunes^26–28^. Furthermore, the comparison of the soil hardness of both habitats showed that the sand fields have harder soils in both depths 10 and 20 cm, in comparison to the habitat of sandy dunes^62^. Both species occurs at sea level^24,26,27^. Two narrow contact zones have been recently described for these species, one on the northern part of the range of *C*. *flamarioni*, in an area extending about 15 km on sand dunes; and the other on the southern part of the range of *C*. *minutus*, in the city of São José do Norte^29^ (see Kubiak *et al*.^29^ for more information; Fig. 7). For the character displacement analysis we only used specimens from areas with sand dunes, where both species present allopatric and sympatric distribution. This habitat is characterized by soft soils and low productivity of plant biomass throughout its entire extension^29^.

### Requirements for testing the geographic predictions of competition exclusion and release

*Ctenomys flamarioni* and *C*. *minutus* meet all the requirements for the test of the geographic predictions of competitive exclusion and release^16^. Thus, the distributions of these species do not broadly overlap, but are either allopatric or parapatric with only two contact zones^23,29^ (Fig. 7). These contact zones are precisely where competitive exclusion could take place. In several sites along most of the distribution of *C*. *flamarioni*, *C*. *minutus* is absent, thus enabling the possibility for competitive release for the former. *Ctenomys minutus* is present in sandy dunes in the northern part of its distribution, where C. *flamarioni* is absent, and therefore competitive release of the former could take place there. Furthermore, despite the small geographic scale of the area in which both species are distributed, the environmental tolerances of the two species significantly differ from each other but show partial overlap. The mean environmental conditions at focal species’ localities significantly differ for seven of a total of 19 bioclimatic variables employed in this study (see Supplementary Table S3). In addition, a Principal Components Analysis showed that the environmental space sampled has conditions in which both species occur (overlap) as well as conditions of exclusivity (occupied by only one of the species)[see Supplementary Fig. S1].

In addition to fulfilling the geographic and environmental requirements for testing the geographic predictions of competitive exclusion and release, both species have similar morphologies, making it likely to have similar requirements and preferences regarding resources in the study region (see Galiano *et al*.^27,30^ and Kubiak *et al*.^29^). Moreover, based on our fieldwork experience, both species seems equally likely to be captured with the same sampling techniques, in compliance with requirements for conducting the tests^16^.

### Data sources

To model the species’ abiotically suitable areas, we used occurrence and climatic data. The use of presence occurrence records (localities) with correct both taxonomic identifications and georeference is critical for satisfactory performance of ecological niche modeling analyses^63–66^. Hence, we gathered occurrence data only from our own observations in the field or from voucher specimens housed at the collection of the *Laboratório de Citogenética e Evolução* of the *Universidade Federal do Rio Grande do Sul* (specimens from our own fieldwork^23,25–27,30,52,67–70^). Several morphological traits permitted unmistakable taxonomic identifications (see Freitas^23^). All localities were georeferenced using a GPS Garmin Vista® device at the exact site of collection or observation. We obtained a total of 74 unique localities, 45 for *C*. *minutus* and 29 for *C*. *flamarioni* (see Supplementary Information). For climatic data, we used 19 WorldClim bioclimatic variables derived from interpolations of precipitation and temperature data, with a resolution of 30 arc-seconds (approximately one kilometer at the Equator; available at^71^). Previous studies have found these variables to be important in determining mammal species distributions (e.g., refs^18,72,73^).

### Model calibrations

In order to defining study regions for model calibrations, we first attempted to use a strategy used in previous studies (e.g.^18,73^), which consists in employing a minimum convex polygons, constructed surrounding large clusters of occurrence records, plus a buffer area outside of this polygon. This operational strategy aims to minimize the inclusion of regions to which the species does not have access to, due to physical barriers or biotic interactions, but that might contain suitable environmental conditions for it. The inclusion of such inaccessible regions would represent a violation to principles for selecting study areas for model calibration (proposed by Anderson and Raza^74^, Barve *et al*.^75^; see also Gutiérrez^66^). Nevertheless, because of the truly narrow, small distributions of our focal species, the study areas that resulted from applying the operational strategy just described were too small and did not include enough environmental heterogeneity for Maxent to characterize the abiotically suitable conditions for each species. To solve this problem, we opted for creating a minimum convex polygon surrounding localities of both species, pooled together, and then delimited a background region by setting a buffer of 50 km around the resulting polygon. The use of an even larger buffer seemed unnecessary because ctenomids have limited both mobility and ability of dispersion^39^.

To model the species’ abiotically suitable areas, we optimized model complexity and predictive power, and conducted model evaluations using a geographically partitioned scheme. The models were constructed with the maximum entropy method implemented in Maxent ver. 3.3.3k^76^, a technique that has performed favorably when compared with analytical alternatives for presence-only data^77–79^. Recent studies have demonstrated the importance of both balancing model complexity and predictive power and evaluating model’s performance with spatially independent data^80–84^. Hence, in order produce the best possible model for each species, avoiding overfitting while maximizing predictive power, we employed the R package ENMeval^81^ to select the optimal combination of two important Maxent’s parameters, the value of the regularization multiplier and the combination of feature classes. We tested regularization multiplier values from 0.5 to 6.0 in increments of 0.5, and the following feature classes (or combinations thereof): (1) linear and quadratic; (2) hinge; (3) linear and hinge; (4) quadratic and hinge; and (5) linear, quadratic, and hinge. ENMeval also allowed us to conduct geographically partitioned evaluations, which we did using the “checkerboard1” data-partitioning scheme—this is a variation of the ‘masked geographically structured’ data-partitioning strategy described in Radosavljevic and Anderson^82^. Model performance was assessed using the Akaike Information Criterion corrected for small sample sizes (AICc^80,82^). For each species, the final model was constructed employing all unique occurrence records and the combination of regularization multiplier and feature classes that produced the lowest AICc value. To assure that the models selected as optimal performed well, we also inspected omission rate and test AUC. The minimum training threshold was used to classify environmental conditions into suitable or not suitable based on model prediction strengths. This classification is a necessary step for the identification of areas of potential sympatry, i.e. those with suitable conditions for both species. The logistic output of Maxent was used for all of the analyses (for details about this format, see Phillips and Dudík^85^).

### Specimens and geometric morphometrics

To test for character displacement we used geometric morphometrics based on skull landmarks. We used a total of 85 skulls of adult specimens collected only in the first-dune line—we did not use skulls from specimens from sandy fields to reduce the environmental bias in the test for character displacements (see below). We analyzed data from 39 skulls of *C*. *flamarioni*, 22 from areas where the other species is absent (hereafter we refer to these sites as ‘areas of allopatry’) and 17 from areas where the other species is present (hereafter we refer to these sites as ‘areas of sympatry’). For *C*. *minutus*, we analyzed data from 46 skulls, 24 from areas of allopatry and 22 from areas of sympatry. All of the specimens are from the *Laboratório de Citogenética e Evolução*, *Departamento de Genética*, *Universidade Federal do Rio Grande do Sul*, Porto Alegre, Brazil (for specimens’ locality data and catalogue numbers see Supplementary Information).

We used standard methods of landmark-based 2D geometric morphometrics to remove non-shape differences among our samples. Skulls ventral view images were taken with a Nikon P100 camera with 13.1 megapixel resolution (3648 × 2736) from a standard distance of 75 mm. On each image, 30 landmarks (see Supplementary Information for landmark positions) were digitized using TpsDig2 software^86^, following Fornel *et al*.^87^. The matrix of landmark coordinates was submitted to a Generalized Procrustes Analysis (GPA) to remove effects not related to shape (position, orientation, and scale^88^). The resulting matrix (i.e. shape variables) was used in a multivariate regression against centroid size to test for the presence of allometry in the samples^89^. Residuals of this analysis were used as a size-corrected shape matrix for all further analyses. The centroid size of each specimen—i.e., the square root of the sum of squared distances of each landmark from the centroid of the configuration—was used as a measure of size^88^. All these procedures were carried out using the software MorphoJ 1.06d^90^. We assumed that sexual dimorphism was negligible for the present purposes, because interspecific differences were almost always greater than the reported sexual dimorphism in both size and shape of the skull^91^, and it has been reported that dimorphism small and constant across geographic locations within species of *Ctenomys* ^87^.

### Tests of the geographic predictions of competitive exclusion and release

We tested for the geographic predictions of competitive exclusion and release based on ENM and occurrence records. In order to identify areas of potential sympatry, we first projected the final model of each species onto geographic space. These projections were made onto a region in the southern Brazilian coastal plain (extent 27–34°N and 47–53°W) that includes the known ranges of both species. We then overlaid the binary predictions of both models, using the same thresholding rule as in the model evaluations (i.e., the minimum training presence threshold). We analyzed the proportions of species localities in areas of potential sympatry along their known contact zones (described in Kubiak *et al*.^29^; see also Fig. 7), directly testing the geographic patterns predicted under competitive exclusion^16^. Under the assumption that competitive exclusion takes place, we expected two possible geographic scenarios (see Gutiérrez *et al*.^18^ for details): first, that one species largely predominates in terms of the proportion of unique localities in actual contact zones (this approach is based on binary maps), or, alternatively, that each species predominates wherever the environmental conditions are more suitable for it than for it putative competitor (this approach is based on maps with continuous values of suitability for each species). We tested for the first of these scenarios using a modification to the method of Anderson *et al*.^16^ proposed by Gutiérrez *et al*.^18^, and which aims to avoid likely biases towards the most broadly distributed species. This modification consists in calculating random expectations values of a binomial test using only unique localities of each species from areas of potential sympatry accessible to both species and, to avoid circularity, excluding those in the actual contact zones. For the second scenario, we examined the areas near the contact zones in more detail, determining for each pixel which species had higher values of predicted suitability (following Gutiérrez *et al*.^18^, Anderson *et al*.^92^).

We also tested for the geographic prediction of competitive release. We inspected areas of potential sympatry out of the known contact zones between the species and expected that, in absence of the putative superior competitor, the putative inferior competitor would inhabit conditions similar to those present in the contact zone.

### Character displacement

We used a one-way analysis of variance (ANOVA) to test size differences between species in sympatry and allopatry (the categorical variable had one factor with four levels: sympatric *C*. *minutus*, allopatric *C*. *minutus*, sympatric *C*. *flamarioni*, and allopatric *C*. *flamarioni*). A Tukey’s test was conducted to verify pairwise size differences. Three measures of size were used independently: centroid size, skull length and body mass. For body mass we used a sub sample of 64 animals that had these records available (22 of sympatric *C*. *minutus*, 15 allopatric *C*. *minutus*, 14 sympatric *C*. *flamarioni*, and 13 allopatric *C*. *flamarioni*; see Supplementary Information). A between-group principal component analysis (BG-PCA) was used to explore patterns of shape variation among these same four groups. The BG-PCA is an alternative to canonical variate analysis (CVA) when the number of individuals is close to the number of variables^93^, as in our case. As herein applied, the BG-PCA consists of a rotation of the shape space in the direction of largest mean group differences, with no distortion of shape distances (as opposed to CVA), and increased ability to discriminate among groups when compared with ordinary PCA^93,94^. We calculated the significance of mean differences between four groups through pairwise tests with 10,000 permutations, using Procrustes distance between means, in the software MorphoJ 1.06d^92^. Because we predicted that specimens of both species have a higher degree of morphological differentiation in sympatry than in allopatry, we tested if the differences of Procrustes distances between the species in sympatry *vs*. allopatry could not be explained due to chance alone. To do this, we calculated the observed difference between the Procrustes distance of the species in sympatry minus the Procrustes distance of species in allopatry. The resulting value indicates how strong is the morphological difference in sympatry relative to that in allopatry. We then used a bootstrap procedure to assign random group labels to each specimen (within species, i.e. random assignment of allopatry or sympatry within each species, but not between them), and re-calculated this difference 1,000 times by chance. We then calculated a *p*-value (at α = 0.05) assessing the number of times that the random difference (derived from bootstrap sets) was equal or greater than the observed difference.

## Electronic supplementary material

supplementary information
