# Supplementary material for: Can Niche Modeling and Geometric Morphometrics Document Competitive Exclusion in a Pair of Subterranean Rodents (Genus Ctenomys) with Tiny Parapatric Distributions?
Source: Sci Rep. 2017 Nov 24;7:16283. doi: 10.1038/s41598-017-16243-2 (PMC5701118; doi:10.1038/s41598-017-16243-2)
Supplement: Supplementary file 1 — supplementary information [file 41598_2017_16243_MOESM1_ESM.pdf]

SUPPLEMENTARY INFORMATION

CAN NICHE MODELING AND GEOMETRIC MORPHOMETRICS DOCUMENT COMPETITIVE EXCLUSION IN A PAIR OF SUBTERRANEAN RODENT (GENUS *CTENOMYS*) WITH TINY PARAPATRIC DISTRIBUTIONS?

Bruno B. Kubiak, Eliécer E. Gutiérrez, Daniel Galiano, Renan Maestri, Thales R. O. de Freitas.

SUPPLEMENTARY INFORMATION 1 – Results of model tuning analyses conducted in ENMeval.

Our analyses to select optimal Maxent's settings identified the use of Linear and Quadratic features as the best combination of features classes for both species. For *C. flamarioni* the best performing value for regularization multiplier was 1, whereas for *C. minutus* it was 1.5. These optimal settings (LQ 1, for *C. flamarioni*; LQ 1.5 for *C. minutus*) showed the lowest mean AICc, which was our primary optimality criterion. They were also the best performing settings considering the mean diff.AUC (i.e. mean training minus testing AUC values), and Mean.ORmin (omission rate calculated with the the minimum training presence threshold). Other settings possessed higher mean AUC values, but the differences were small. See tables with values for all evaluation metrics below.

Table S 1. Results from tuning experiments using ENMeval for *Ctenomys flamarioni*. Matrix of evaluation criteria sorted by AICc, the optimality criterion used for model tuning in this study. The combination of feature class and regularization multiplier with the lowest AICc was considered the 'best' model and used for final model calibration and all subsequent analysis; here the best combination of settings were Linear and Quadratic and regularization multiplier = 3. The default settings (Linear, Quadratic, and Hinge; regularization = 1) led to models that were substantially worse. Mean omission rate was calculated using the minimum training presence threshold.

Feature Class	Regularization Multiplier	Mean Test AUC	Variance Test AUC	Mean AUC DIFF	Variance AUC DIFF	Mean Omission Rate	Variance Omission Rate	AICc	Delta AICc	Model Parameters
H	0.5	0.94837	0.00001	0.03474	0.00001	0.26136	0.00013	NA	NA	50
QH	0.5	0.94694	0.00001	0.03604	0.00001	0.35227	0.01046	NA	NA	33
LQ	0.5	0.93494	0.00006	0.02648	0.00023	0.24432	0.01424	529.0159	31.54638421	14
LH	0.5	0.94839	0.00000	0.03440	0.00001	0.26136	0.00013	NA	NA	37
LQH	0.5	0.94654	0.00000	0.03624	0.00001	0.26136	0.00013	NA	NA	45
H	1	0.93853	0.00011	0.02234	0.00025	0.18182	0.03306	563.586132	66.11661619	17
QH	1	0.94017	0.00003	0.02007	0.00014	0.18182	0.03306	558.6894797	61.21996396	17
**LQ**	**1**	**0.93188**	**0.00013**	**0.01847**	**0.00020**	**0.13636**	**0.01860**	**497.4695158**	**0**	**7**
LH	1	0.93765	0.00013	0.02291	0.00027	0.18182	0.03306	580.5217055	83.05218972	18
LQH	1	0.93956	0.00004	0.02082	0.00016	0.18182	0.03306	578.0532946	80.58377887	18
H	1.5	0.92347	0.00041	0.02563	0.00066	0.13636	0.01860	516.8429289	19.37341316	11
QH	1.5	0.93121	0.00011	0.01770	0.00015	0.13636	0.01860	509.624857	12.15534122	10
LQ	1.5	0.92921	0.00020	0.01676	0.00028	0.13636	0.01860	502.4284193	4.958903501	7
LH	1.5	0.92706	0.00028	0.02204	0.00049	0.13636	0.01860	523.1460541	25.67653837	12
LQH	1.5	0.93040	0.00012	0.01910	0.00018	0.13636	0.01860	509.5695492	12.10003346	10
H	2	0.91645	0.00057	0.02767	0.00077	0.13636	0.01860	519.8563936	22.38687784	10
QH	2	0.93001	0.00018	0.01504	0.00023	0.09091	0.00826	513.0998754	15.63035959	9
LQ	2	0.92781	0.00021	0.01673	0.00028	0.09091	0.00826	503.1318155	5.662299742	6
LH	2	0.92216	0.00033	0.02212	0.00049	0.13636	0.01860	524.6147077	27.14519193	11
LQH	2	0.92840	0.00023	0.01673	0.00028	0.09091	0.00826	513.2646974	15.79518165	9
H	2.5	0.90936	0.00064	0.02880	0.00083	0.09091	0.00826	522.1891694	24.71965364	9

Feature Class	Regularization Multiplier	Mean Test AUC	Variation Test AUC	Mean AUC DIFF	Variation AUC DIFF	Mean Omission Rate	Variation Omission Rate	AICc	Delta AICc	Model Parameters
QH	2.5	0.92746	0.00017	0.01527	0.00023	0.09091	0.00826	506.3315215	8.862005717	6
LQ	2.5	0.92577	0.00021	0.01714	0.00029	0.09091	0.00826	506.3322411	8.862725321	6
LH	2.5	0.91800	0.00028	0.02112	0.00045	0.09091	0.00826	515.6319032	18.16238746	8
LQH	2.5	0.92586	0.00021	0.01714	0.00029	0.09091	0.00826	506.3337549	8.864239119	6
H	3	0.90571	0.00055	0.02624	0.00069	0.09091	0.00826	519.4917576	22.02224186	7
QH	3	0.92490	0.00013	0.01539	0.00024	0.09091	0.00826	509.2339791	11.76446333	6
LQ	3	0.92262	0.00017	0.01752	0.00031	0.09091	0.00826	509.207253	11.73773724	6
LH	3	0.91376	0.00024	0.02007	0.00040	0.09091	0.00826	510.9101229	13.44060708	6
LQH	3	0.92301	0.00018	0.01752	0.00031	0.09091	0.00826	509.1947311	11.72521534	6
H	3.5	0.90222	0.00051	0.02564	0.00066	0.09091	0.00826	519.2173194	21.74780358	6
QH	3.5	0.92304	0.00009	0.01446	0.00021	0.09091	0.00826	508.3388378	10.86932202	5
LQ	3.5	0.91941	0.00011	0.01712	0.00029	0.09091	0.00826	508.3387823	10.86926654	5
LH	3.5	0.91013	0.00022	0.01917	0.00037	0.09091	0.00826	509.5815466	12.11203086	5
LQH	3.5	0.92077	0.00014	0.01712	0.00029	0.09091	0.00826	508.3420784	10.87256262	5
H	4	0.90057	0.00043	0.02409	0.00058	0.09091	0.00826	515.9500787	18.48056294	4
QH	4	0.92044	0.00007	0.01441	0.00021	0.09091	0.00826	509.7825473	12.31303151	5
LQ	4	0.91648	0.00005	0.01582	0.00025	0.09091	0.00826	509.7793672	12.30985144	5
LH	4	0.90598	0.00023	0.01975	0.00039	0.09091	0.00826	511.1268383	13.65732255	5
LQH	4	0.91912	0.00009	0.01582	0.00025	0.09091	0.00826	509.7831384	12.31362262	5
H	4.5	0.89659	0.00037	0.02383	0.00057	0.09091	0.00826	521.8822364	24.41272066	5
QH	4.5	0.91651	0.00004	0.01441	0.00021	0.09091	0.00826	511.2517444	13.78222866	5
LQ	4.5	0.91310	0.00002	0.01542	0.00024	0.09091	0.00826	511.2517792	13.78226341	5
LH	4.5	0.90181	0.00020	0.01993	0.00040	0.09091	0.00826	512.7184373	15.24892157	5
LQH	4.5	0.91560	0.00005	0.01542	0.00024	0.09091	0.00826	511.2521696	13.78265378	5
H	5	0.89381	0.00036	0.02327	0.00054	0.09091	0.00826	524.7994548	27.32993903	5
QH	5	0.91288	0.00002	0.01444	0.00021	0.09091	0.00826	509.6667252	12.19720944	4
LQ	5	0.91064	0.00002	0.01521	0.00023	0.09091	0.00826	509.666575	12.19705926	4
LH	5	0.89802	0.00021	0.01885	0.00036	0.09091	0.00826	511.254697	13.78518123	4
LQH	5	0.91187	0.00003	0.01521	0.00023	0.09091	0.00826	509.6665806	12.19706481	4
H	5.5	0.89488	0.00032	0.02060	0.00042	0.09091	0.00826	524.2352078	26.76569206	4
QH	5.5	0.91054	0.00002	0.01452	0.00021	0.09091	0.00826	508.3143847	10.84486893	3
LQ	5.5	0.90900	0.00001	0.01483	0.00022	0.09091	0.00826	508.3143943	10.84487852	3
LH	5.5	0.89691	0.00025	0.01772	0.00031	0.09091	0.00826	510.0263267	12.5568109	3
LQH	5.5	0.91014	0.00002	0.01483	0.00022	0.09091	0.00826	508.3143989	10.84488309	3
H	6	0.89642	0.00026	0.01710	0.00029	0.09091	0.00826	526.5701355	29.10061971	4
QH	6	0.90822	0.00002	0.01477	0.00022	0.09091	0.00826	509.6387042	12.16918838	3
LQ	6	0.90822	0.00002	0.01477	0.00022	0.09091	0.00826	509.6387113	12.16919549	3

Feature Class	Regularization Multiplier	Mean Test AUC	Variation Test AUC	Mean AUC DIFF	Variation AUC DIFF	Mean Omission Rate	Variation Omission Rate	AICc	Delta AICc	Model Parameters
LH	6	0.89747	0.00023	0.01588	0.00025	0.09091	0.00826	511.4667886	13.99727277	3
LQH	6	0.90822	0.00002	0.01477	0.00022	0.09091	0.00826	509.6387035	12.16918777	3
H	6.5	0.89710	0.00024	0.01556	0.00024	0.09091	0.00826	526.2050644	28.73554859	3
QH	6.5	0.90844	0.00004	0.01465	0.00021	0.09091	0.00826	513.767408	16.29789221	4
LQ	6.5	0.90740	0.00003	0.01465	0.00021	0.09091	0.00826	513.7687181	16.29920232	4
LH	6.5	0.90120	0.00039	0.01556	0.00024	0.09091	0.00826	512.9377918	15.468276	3
LQH	6.5	0.90699	0.00003	0.01472	0.00022	0.09091	0.00826	513.7677093	16.29819348	4
H	7	0.89710	0.00024	0.01556	0.00024	0.09091	0.00826	528.6896644	31.22014864	3
QH	7	0.90635	0.00004	0.01422	0.00020	0.09091	0.00826	512.2939445	14.8244287	3
LQ	7	0.90635	0.00004	0.01422	0.00020	0.09091	0.00826	512.2939254	14.82440959	3
LH	7	0.89710	0.00024	0.01556	0.00024	0.09091	0.00826	514.416428	16.94691221	3
LQH	7	0.90589	0.00005	0.01440	0.00021	0.09091	0.00826	512.2939345	14.82441876	3
H	7.5	0.89710	0.00024	0.01556	0.00024	0.09091	0.00826	528.4610819	30.99156611	2
QH	7.5	0.90455	0.00007	0.01445	0.00021	0.09091	0.00826	513.5224976	16.05298183	3
LQ	7.5	0.90455	0.00007	0.01445	0.00021	0.09091	0.00826	513.5224935	16.05297776	3
LH	7.5	0.89710	0.00024	0.01556	0.00024	0.09091	0.00826	515.9202609	18.45074511	3
LQH	7.5	0.90455	0.00007	0.01446	0.00021	0.09091	0.00826	513.5226606	16.05314481	3
H	8	0.89710	0.00024	0.01556	0.00024	0.09091	0.00826	530.7013669	33.23185111	2
QH	8	0.90270	0.00010	0.01491	0.00022	0.09091	0.00826	514.7650402	17.29552442	3
LQ	8	0.90270	0.00010	0.01491	0.00022	0.09091	0.00826	514.7650603	17.29554456	3
LH	8	0.89710	0.00024	0.01556	0.00024	0.09091	0.00826	517.4412625	19.97174673	3
LQH	8	0.90270	0.00010	0.01492	0.00022	0.09091	0.00826	514.7650317	17.29551596	3

Table S2. Results from tuning experiments using ENMeval for *Ctenomys minutus*. Matrix of evaluation criteria sorted by AICc, the optimality criterion used for model tuning in this study. The combination of feature class and regularization multiplier with the lowest AICc was considered the 'best' model and used for final model calibration and all subsequent analysis; here the best combination of settings were Linear and Quadratic and regularization multiplier = 3. The default settings (Linear, Quadratic, and Hinge; regularization = 1) led to models that were substantially worse. Mean omission rate was calculated using the minimum training presence threshold.

Feature Class	Regularization Multiplier	Mean Test AUC	Variance Test AUC	Mean AUC DIFF	Variance AUC DIFF	Mean Omission Rate	Variance Omission Rate	AICc	Delta AICc	Model Parameters
H	0.5	0.90785	0.00003	0.03632	0.00015	0.24741	0.00324	NA	NA	43
QH	0.5	0.91125	0.00009	0.03368	0.00021	0.24741	0.00324	NA	NA	43
LQ	0.5	0.89295	0.00046	0.01800	0.00032	0.02174	0.00047	876.3307155	0.609794966	14
LH	0.5	0.90962	0.00007	0.03445	0.00017	0.20393	0.00018	NA	NA	44
LQH	0.5	0.91121	0.00009	0.03302	0.00021	0.22567	0.00124	NA	NA	46
H	1	0.88804	0.00015	0.02497	0.00034	0.04762	0.00227	991.8439511	116.1230306	28
QH	1	0.89049	0.00025	0.02244	0.00050	0.06936	0.00067	1029.437882	153.7169616	30
LQ	1	0.88192	0.00033	0.01860	0.00035	0.02174	0.00047	879.018572	3.297651506	11
LH	1	0.88694	0.00020	0.02199	0.00046	0.04762	0.00227	1081.59954	205.8786192	32
LQH	1	0.89068	0.00022	0.02224	0.00049	0.06936	0.00067	1028.3259	152.6049799	30
H	1.5	0.87926	0.00037	0.01935	0.00037	0.02381	0.00057	944.5806888	68.85976822	23
QH	1.5	0.87947	0.00030	0.02175	0.00047	0.04555	0.00000	890.8727757	15.15185514	16
**LQ**	**1.5**	**0.87441**	**0.00032**	**0.01834**	**0.00034**	**0.02174**	**0.00047**	**875.7209205**	**0**	**7**
LH	1.5	0.87783	0.00029	0.02116	0.00045	0.04555	0.00000	902.2065736	26.48565307	18
LQH	1.5	0.87996	0.00029	0.02114	0.00045	0.04555	0.00000	908.6964734	32.97555286	19
H	2	0.87779	0.00054	0.01947	0.00038	0.02381	0.00057	888.2566675	12.53574694	12
QH	2	0.87219	0.00028	0.02169	0.00047	0.04555	0.00000	895.8804474	20.15952689	15
LQ	2	0.86997	0.00033	0.01929	0.00037	0.02174	0.00047	876.7125293	0.991608729	5
LH	2	0.87550	0.00035	0.02128	0.00045	0.02381	0.00057	900.1935018	24.47258123	16
LQH	2	0.87225	0.00028	0.02170	0.00047	0.04555	0.00000	895.7805567	20.05963618	15
H	2.5	0.87493	0.00063	0.01906	0.00036	0.02381	0.00057	892.2575501	16.53662955	12
QH	2.5	0.86578	0.00045	0.02199	0.00048	0.04555	0.00000	896.6624395	20.94151901	14
LQ	2.5	0.86301	0.00041	0.01964	0.00039	0.02174	0.00047	879.897867	4.176946462	5
LH	2.5	0.87496	0.00042	0.02007	0.00040	0.04555	0.00000	888.4234946	12.70257408	12

Feature Class	Regularization Multiplier	Mean Test AUC	Variation Test AUC	Mean AUC DIFF	Variation AUC DIFF	Mean Omission Rate	Variation Omission Rate	AICc	Delta AICc	Model Parameters
LQH	2.5	0.86693	0.00042	0.02162	0.00047	0.04555	0.00000	896.4571878	20.73626731	14
H	3	0.87088	0.00060	0.01819	0.00033	0.02381	0.00057	895.4892945	19.76837396	12
QH	3	0.86473	0.00056	0.02046	0.00042	0.04555	0.00000	896.2441842	20.52326362	13
LQ	3	0.85966	0.00041	0.01933	0.00037	0.02174	0.00047	882.3332969	6.61237632	5
LH	3	0.87365	0.00047	0.01900	0.00036	0.04555	0.00000	888.2856446	12.5647241	11
LQH	3	0.86523	0.00054	0.02021	0.00041	0.04555	0.00000	896.258926	20.53800544	13
H	3.5	0.86492	0.00064	0.01823	0.00033	0.00000	0.00000	891.2730418	15.55212126	10
QH	3.5	0.86299	0.00058	0.01935	0.00037	0.04555	0.00000	892.3780476	16.65712709	11
LQ	3.5	0.85701	0.00036	0.01833	0.00034	0.02174	0.00047	886.7619543	11.04103377	6
LH	3.5	0.86970	0.00047	0.01857	0.00034	0.02174	0.00047	891.8372557	16.11633521	11
LQH	3.5	0.86361	0.00056	0.01914	0.00037	0.04555	0.00000	899.6045853	23.8836648	13
H	4	0.85817	0.00081	0.01834	0.00034	0.00000	0.00000	901.8511442	26.13022363	12
QH	4	0.85942	0.00049	0.01821	0.00033	0.02174	0.00047	884.9717473	9.250826778	8
LQ	4	0.85446	0.00030	0.01786	0.00032	0.02174	0.00047	886.0142458	10.2933253	5
LH	4	0.86586	0.00046	0.01808	0.00033	0.04348	0.00189	887.850229	12.12930845	9
LQH	4	0.86023	0.00047	0.01813	0.00033	0.02174	0.00047	887.9186091	12.19768854	9
H	4.5	0.85323	0.00083	0.01862	0.00035	0.00000	0.00000	894.1940607	18.47314021	9
QH	4.5	0.85661	0.00042	0.01741	0.00030	0.02174	0.00047	890.9857329	15.26481239	9
LQ	4.5	0.85335	0.00026	0.01733	0.00030	0.02174	0.00047	886.791698	11.07077746	5
LH	4.5	0.86475	0.00043	0.01740	0.00030	0.04348	0.00189	887.3947532	11.67383269	8
LQH	4.5	0.85760	0.00042	0.01733	0.00030	0.02174	0.00047	894.1011084	18.38018786	10
H	5	0.85092	0.00073	0.01848	0.00034	0.00000	0.00000	908.3273434	32.60642288	12
QH	5	0.85503	0.00037	0.01683	0.00028	0.02174	0.00047	894.1595066	18.43858606	9
LQ	5	0.85309	0.00027	0.01681	0.00028	0.02174	0.00047	890.5608819	14.83996133	6
LH	5	0.86313	0.00040	0.01702	0.00029	0.04348	0.00189	887.2458597	11.52493917	7
LQH	5	0.85571	0.00036	0.01681	0.00028	0.02174	0.00047	893.7177553	17.99683475	9
H	5.5	0.84966	0.00067	0.01784	0.00032	0.00000	0.00000	908.4147147	32.69379419	11
QH	5.5	0.85362	0.00032	0.01637	0.00027	0.02174	0.00047	894.5227855	18.80186495	8
LQ	5.5	0.85314	0.00027	0.01630	0.00027	0.02174	0.00047	892.12601	16.40508946	6
LH	5.5	0.86203	0.00038	0.01671	0.00028	0.04348	0.00189	890.3461978	14.62527722	7
LQH	5.5	0.85441	0.00031	0.01630	0.00027	0.02174	0.00047	897.0715163	21.35059581	9
H	6	0.84502	0.00062	0.01776	0.00032	0.00000	0.00000	908.7867485	33.06582799	10
QH	6	0.85244	0.00029	0.01643	0.00027	0.02174	0.00047	894.7017716	18.98085108	7
LQ	6	0.85334	0.00027	0.01608	0.00026	0.02174	0.00047	893.7659183	18.04499772	6
LH	6	0.86073	0.00035	0.01651	0.00027	0.04348	0.00189	893.1403298	17.41940923	7
LQH	6	0.85330	0.00027	0.01611	0.00026	0.02174	0.00047	894.1341817	18.41326118	7
H	6.5	0.84195	0.00061	0.01823	0.00033	0.00000	0.00000	902.8460284	27.12510786	7

Feature Class	Regularization Multiplier	Mean Test AUC	Variation Test AUC	Mean AUC DIFF	Variation AUC DIFF	Mean Omission Rate	Variation Omission Rate	AICc	Delta AICc	Model Parameters
QH	6.5	0.85256	0.00029	0.01640	0.00027	0.02174	0.00047	897.8872356	22.16631503	7
LQ	6.5	0.85330	0.00027	0.01619	0.00026	0.02174	0.00047	895.4272301	19.70630956	6
LH	6.5	0.85987	0.00036	0.01655	0.00027	0.04348	0.00189	890.02867	14.30774942	5
LQH	6.5	0.85331	0.00027	0.01616	0.00026	0.02174	0.00047	897.2604871	21.53956652	7
H	7	0.83684	0.00080	0.01947	0.00038	0.00000	0.00000	903.5956801	27.87475952	6
QH	7	0.85267	0.00029	0.01633	0.00027	0.02174	0.00047	898.0100836	22.28916303	6
LQ	7	0.85330	0.00028	0.01630	0.00027	0.02174	0.00047	897.1707226	21.44980209	6
LH	7	0.85741	0.00038	0.01644	0.00027	0.04348	0.00189	892.5178036	16.79688311	5
LQH	7	0.85330	0.00028	0.01634	0.00027	0.02174	0.00047	897.1694777	21.44855716	6
H	7.5	0.83553	0.00073	0.01947	0.00038	0.00000	0.00000	904.4153554	28.69443486	5
QH	7.5	0.85275	0.00029	0.01620	0.00026	0.02174	0.00047	899.7955127	24.07459212	6
LQ	7.5	0.85340	0.00028	0.01615	0.00026	0.02174	0.00047	898.9889768	23.26805623	6
LH	7.5	0.85554	0.00038	0.01645	0.00027	0.04348	0.00189	893.7690877	18.04816717	5
LQH	7.5	0.85341	0.00028	0.01616	0.00026	0.02174	0.00047	899.1378701	23.4169496	6
H	8	0.83443	0.00067	0.01947	0.00038	0.00000	0.00000	904.76157	29.04064945	4
QH	8	0.85289	0.00029	0.01614	0.00026	0.02174	0.00047	901.7766302	26.05570969	6
LQ	8	0.85341	0.00028	0.01608	0.00026	0.02174	0.00047	898.0328373	22.31191681	5
LH	8	0.85413	0.00036	0.01629	0.00027	0.04348	0.00189	895.05597	19.33504948	5
LQH	8	0.85342	0.00028	0.01607	0.00026	0.02174	0.00047	898.0569498	22.33602927	5

SUPPLEMENTARY INFORMATION 2

Table S3. Results from Wilcoxon test comparing pairs of means of bioclimatic variables at localities between the focal species. This non-parametric test was conducted after a Shapiro test demonstrated that most variables are not normally distributed. The analysis was carried out in R employing its native function "*wilcox.test*" at a confidence level of 0.5. Means were significantly different for seven out of 19 bioclimatic variables. Asterisk denotes significantly different values. The meanings of the codes of each bioclimatic variable follow below the table.

**Variable**	***C. flamarioni***	***C. minutus***	**W**	***p*-value**
bio1	184.19	188.20	416	0.3632
bio2	76.37	79.39	530	0.004212*
bio3	43.89	45.16	377.5	0.8198
bio4	3315.63	3240.36	433.5	0.236
bio5	272.96	276.66	601	3.85E-05*
bio6	100.70	102.66	312	0.3651
bio7	172.26	174.00	480	0.04588*
bio8	152.52	169.66	337.5	0.6453
bio9	202.48	196.50	557.5	0.0007996*
bio10	227.44	230.43	615	9.68E-06*
bio11	142.41	147.48	339.5	0.671
bio12	1379.74	1421.84	423	0.3155
bio13	136.22	142.98	436.5	0.2134
bio14	91.63	92.43	333.5	0.5974
bio15	11.59	12.91	450	0.1373
bio16	380.89	401.11	526	0.005193*
bio17	302.15	305.07	355	0.8762
bio18	347.37	370.05	356.5	0.8967
bio19	369.30	366.77	612	1.89E-05*

Bioclimatic variables are coded as follows: bio1 – Annual mean temperature; bio2 – Mean diurnal range (mean of monthly (max temp - min temp); bio3 – Isothermality (bio2/bio7) (*100); bio4 – Temperature Seasonality (standard deviation *100); bio5 – Max temperature of warmest month; bio6 – Min temperature of coldest month; bio7 – Temperature annual range (bio5-bio6); bio8 – Mean temperature of wettest quarter; bio9 – Mean temperature of driest quarter; bio10 – Mean temperature of warmest quarter; bio11 – Mean temperature of coldest quarter; bio12 – Annual precipitation; bio13 – Precipitation of wettest month; bio14 – Precipitation of driest month; bio15 – Precipitation seasonality (coefficient of variation); bio16 – Precipitation of wettest quarter; bio17 – Precipitation of driest quarter; bio18 – Precipitation of warmest quarter; bio19 – Precipitation of coldest quarter.

SUPPLEMENTARY INFORMATION 3

Figure S1. Climatic variability among localities of focal species. Locality scores are plotted on the first two components resulting from a principal component analysis based on natural logarithms of values of bioclimatic variables at localities of focal species. Circles represent localities of *Ctenomys flamarioni* and triangles those of *C. minutus*. The analysis was based on the correlation matrix using the native function of R "*princomp*".

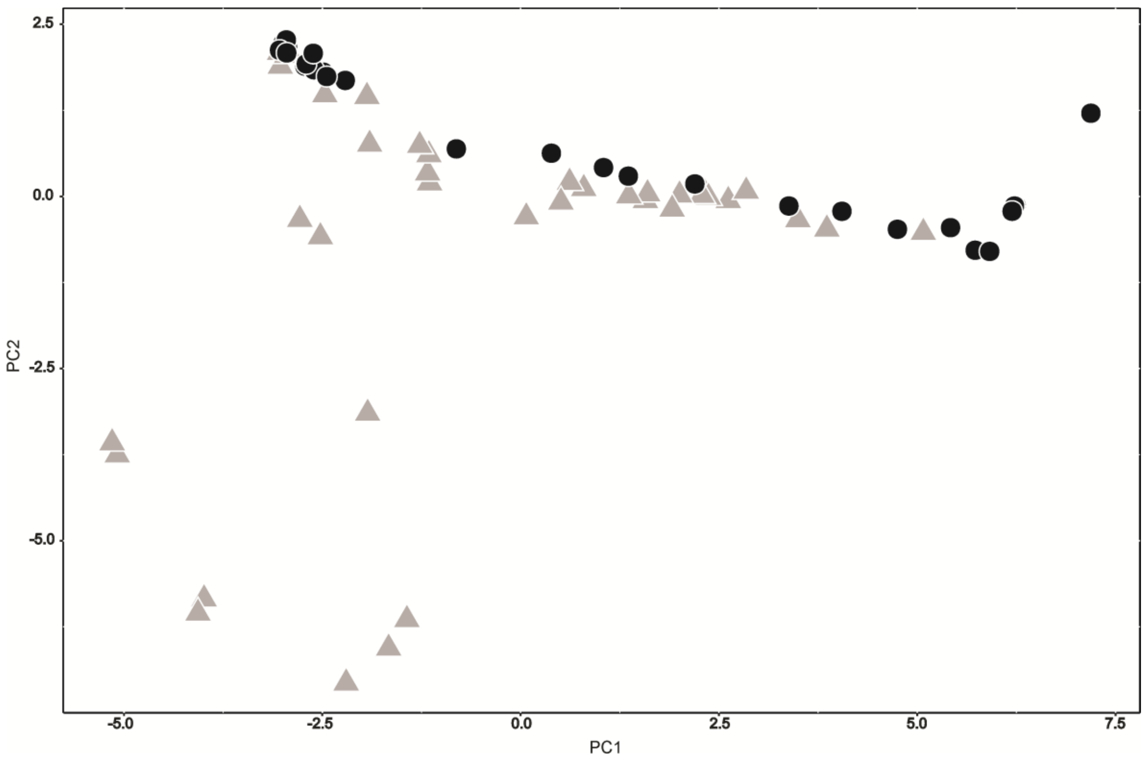

SUPPLEMENTARY INFORMATION 4

Figure S2. Typical habitats of the coastal plain of southern Brazil. The sandy dunes is habitated by *C. flamarioni* and *C. minutus*, whereas only *C. minutus* inhabits the sandy fields.

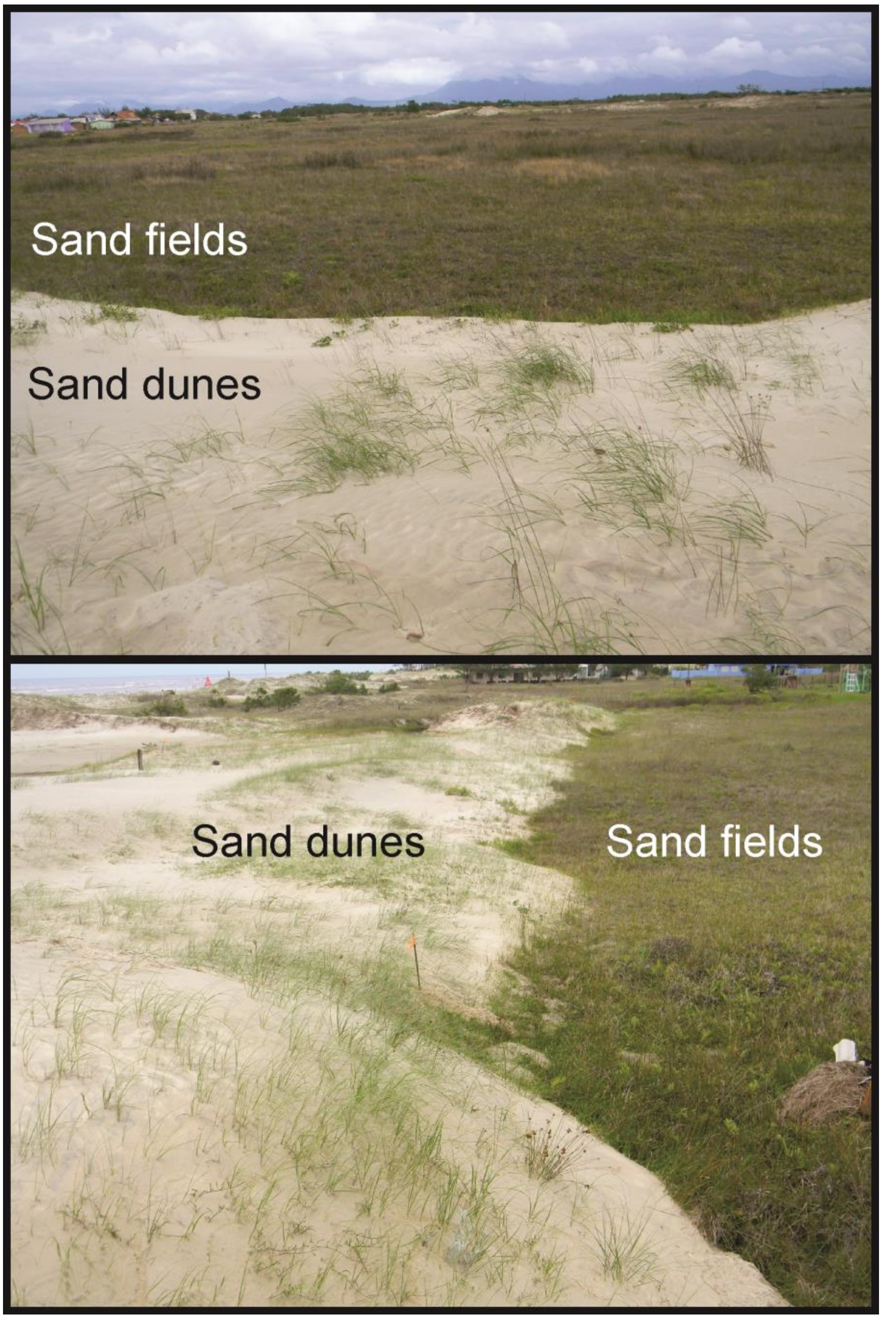

SUPPLEMENTARY INFORMATION 5 - Below we list the localities of *Ctenomys flamarioni* and *C. minutus* used in this study (Fig. 1). All locations are located in the Brazilian states of Rio Grande do Sul (RS) and Santa Catarina (SC).

Ctenomys flamarioni - 27 unique georeferenced localities: 1. Praia do Barco/RS (29º43'41"S, 50º00'18"W), TR 1626; 2. Praia do Barco/RS (29º42'11"S, 49º59'13"W), TR 1853; 3. Imbé/RS (29º57'11"S, 50º06'50"W), TR 2812; 4. Albatroz/RS (29º53'35"S, 50º05'20"W), TR 2819; 5. Santa Terezinha do Norte/RS (29º52'55"S, 50º04'34"W), TR 050; 6. Atlântida Sul/RS (29º52'36"S, 50º05'06"W), TR1919; 7. Mariapolis/RS (29º50'42"S, 50º 04' 19"W), TR 1584; 8.ESEC Taim/RS (32º43'44"S, 52º27'18"W), TR 1721; 9. ESEC Taim/RS (32º43'16"S, 52º27'14"W), TR 1736; 10. Atlântida/RS (29º46'41"S, 50º01'48"W), TR 048; 11. Xangri-lá/RS (29º48'43"S, 50º02'46"W), TR 056; 12. Praia da harmonia/RS (29º54'40"S, 50º05'53"), TR 1535; 13. Magistério/RS (30º17'24"S, 50º15'32"W), TR 029; 14. Vila São Simão/RS (30º57'43"S, 50º40'48"W), TR 215; 15. Bojuru/RS (31º38'42"S, 51º22'19"W), TR 203; 16. Praia do Mar Grosso/RS (32º02'54"S, 51º59'14"W), TR 2213; 17. Cassino (32º09'40"S, 52º06'47"W), TR 500; 18. Santa Vitória do Palmar/RS (32º42'14"S, 52º27'11"W), TR 474; 19. Barra do Chuí/RS (33º43'41"S, 53º21'50"W), TR 062; 20. Praia do Cassino/RS (32º22'50"S, 52º19'25"W), TR 689; 21. São José do Norte (31º55'12"S, 51º51'18"W), TR 2215; 22. Farol da Conceição (31º47'17"S, 51º35'46"W), TR 2192; 23. Mostardas (31º15'23"S, 50º54'59"W), TR 203; 24. Farol da Solidão/RS (30º47'42"S, 50º33'18"W), TR 2074; 25. Lagoa Bacopari (30º32'13"S, 50º22'19"W), TR 213; 26. Praia do Barco (29º42'58"S, 49º59'46"W), TR 499; 27. Praia do Barco (29º41'10"S, 49º58'48"W), TR 1618.

Ctenomys minutus: 45 unique georeferenced localities: 1. São José do Norte/RS (31º57'21S, 51º55'54"W), TR 2224; 2. Bojuru/RS (20 Km South) (31º43'59"S, 51º34'00"W), TR 2227; 3. Bojuru/RS (31º37'59"S, 51º25'59"W), TR 2239; 4.

Tavares/RS (31º22'59"S, 51º09'W), TR 1941; 5. Tavares/RS (31º16'59"S, 51º04'59"W), TR 2089; 6. Mostardas/RS (26 Km South) (31º15'46"S, 51º02'34"W), TR 1099; 7. Mostardas/RS (17 Km South) (31º14'30"S, 51º01'12"W), TR 1091; 8. TR 688 – Mostardas/RS – 31º08'48"S, 50º56'11"W; 9. Mostardas/RS (Km 115) (31º08'48"S, 50º56'11"W), TR 852; 10. BR 101 – KM108/RS (31º08'48"S, 50º56'11"W), TR 856; 11. Mostardas/RS (Lagoa das Figueiras) (30º59'36"S, 50º49'27"W), TR 847; 12. BR 101 – KM101/RS (30°56'55"S, 50°46'26"W), TR 199; 13. Capivaria do Sul/RS (30º54'45"S, 50º44'16"W), TR 848; 14. Mostardas/RS (Fazenda Ressaca – KM 96) (30º40'59"S, 50º33'22"W), TR 845; 15. Mostardas/RS (Granja Passo Fundo) (30°36'01"S, 50º29'40"W), TR 840; 16. BR101 – KM35/RS (30º27'10"S, 50º29'40"W), TR 836; 17. Palmares do Sul/RS – (30º15'22"S, 50º28'49"W), TR 2040; 18. BR101 – KM35/RS – (30º27'10"S, 50º29'40"W), TR 839; 19. Palmares do Sul/RS (30º15'22"S, 50º28'49"W), TR 2092; 20. Pitangueira/RS (30º03'11"S, 50º22'38"W), TR 442; 21. Passinhos/RS (30º01'24"S, 50º22'45"W), TR 2035; 22. Lagoa dos Barros/RS (29º55'27"S, 50º19'05"W), TR 209; 23. Lagoa dos Barros/RS (29º59'44"S, 50º22'20"W), TR 407; 24. Lagoa Fortaleza/RS (30º09'37"S, 50º13'36"W), TR 2002; 25. Lagoa Suzana/RS (30°09'23"S, 50º15'58"W), TR 453; 26. Lagoa Fortaleza/RS (30º09'37"S, 50º13'36"W), TR 2102; 27. Nova Tramandaí/RS (29º59'06"S 50º12'27"W), TR 166; 28. Lagoa Emboaba/RS (29º58'28"S, 50º12'26"W), TR 020; 29. Tramandaí/RS (29º51'08"S, 50º12'05"W), TR 033; 30. Lagoa Emboaba/RS (29º58'28"S, 50º12'26"W), TR 023; 31. Tramandaí/RS (lagoa Traíras) (29º51'08"S, 50º12'05"W), TR 039; 32. Praia do Barco/RS (29º42'26"S, 49º59'03"W), TR 2814; 33. Praia do Barco/RS (29º42'22"S, 49º58'58") TR 2815; 34. Praia do Barco/RS (29º40'53"S 49º58'08"W), TR 1620; 35. Torres/RS (29º21'12"S, 49º43'56"W), TR 2347; 36. Passo de Torres/SC (29º19'04"S, 49º42'45"W), TR 212; 37. Praia da Gaivota/SC (29º09'21"S,

49º34'27"W), TR 2379; 38. Morro dos Conventos/SC (28º56'38"S, 49º21'50"W), TR 1216; 39. Ilhas/SC (28º54'25"S, 49º20'27"W), TR 1166; 40. Lagoa dos Esteves/SC (28º50'15"S, 49º16'45"W), TR 1170; 41. Balneário Arroio Corrente/SC (28º41'56"S, 49º01'26"W), TR 004; 42. Jaguaruna/SC (28º39'29"S, 49º00'46"W), TR 007; 43. Praia de Jaguaruna/SC (28º41'56"S, 49º01'26"W), TR 1164; 44. Farol de Santa Marta/SC (28º35'53"S, 48º50'19"W), TR 1200; 45. Farol de Santa Marta/SC (28º35'49"S, 48º49'08"W), TR 1214.

SUPPLEMENTARY INFORMATION 6 – Below we list the specimens' locality data, catalogue numbers, body mass and skull length of *Ctenomys flamarioni* and *C. minutus* used in geometric morphometric analysis. All locations are located in the Brazilian states of Rio Grande do Sul (RS) and Santa Catarina (SC).

C. flamarioni - Allopatry			
ID	Body mass (g)	Skull lenght (mm)	Locality
PUC 39	-	547	Tramandaí/RS
PUC 46	239	448	Tramandaí/RS
PUC 80	-	503	Praia do Cassino/RS
PUC 82	-	554	Praia do Cassino/RS
PUC 83	-	554	Praia do Cassino/RS
PUC 232	283	563	Praia rei do peixe, Palmares/RS
PUC 384	-	585	Farol da Solidão/RS
PUC 613	198	521	Rainha do Mar/RS
TR 30	250	515	Cidreira/RS
TR 32	200	546	Cidreira/RS
TR 47	300	470	Santa Terezinha/RS
TR 61	330	502	Fazenda Caçapava, Taim/RS
TR 62	208	528	Fazenda Caçapava, Taim/RS
TR 64	147	536	Fazenda Caçapava, Taim/RS
TR 65	-	504	Fazenda Caçapava, Taim/RS
TR 66	-	536	Fazenda Caçapava, Taim/RS
TR 67	-	510	Fazenda Caçapava, Taim/RS
TR 230	155	462	Farol da Solidão/RS
TR 485	292	505	Tramandaí/RS
TR 494	310	554	São Simão/RS
TR 495	210	529	Fazenda Caçapava, Taim/RS
TR 500	-	490	Praia do Cassino/RS
C. flamarioni - Sympatry			
ID	Body mass (g)	Skull lenght (mm)	Locality
PUC 45	178	544	Arroio Teixeira/RS
PUC 47	219	544	Capão Novo/RS
TR 44	250	482	Praia do Barco/RS
TR 45	250	476	Praia do Barco/RS
TR 54	180	479	Praia do Barco/RS
TR 1842	310	503	Praia do Barco/RS
TR 1845	215	502	Praia do Barco/RS
TR 1859	230	514	Praia do Barco/RS
TR 1852	-	525	Praia do Barco/RS
TR 1853	-	513	Praia do Barco/RS
TR 1849	-	558	Praia do Barco/RS
TR 1861	345	504	Praia do Barco/RS
TR 1863	210	568	Praia do Barco/RS
TR 1864	245	566	Praia do Barco/RS
TR 1865	305	519	Praia do Barco/RS
TR 1866	195	544	Praia do Barco/RS

TR 1867	245	521	Praia do Barco/RS
C. minutus - Allopatry			
ID	Body mass (g)	Skull lenght (mm)	Locality
PUC 49	178	519	Praia da Cal, Torres/RS
PUC 281	194	566	Praia de Jaguaruna/SC
PUC 288	197	490	Praia de Jaguaruna/SC
PUC 290	-	458	Praia de Jaguaruna/SC
PUC 291	206	530	Praia de Jaguaruna/SC
PUC 293	250	535	Praia de Jaguaruna/SC
PUC 312	298	497	Passo de Torres/SC
PUC 313	220	538	Passo de Torres/SC
PUC 414	-	491	Morro dos Conventos/RS
PUC 426	-	513	Morro dos Conventos/RS
PUC 427	-	445	Morro dos Conventos/RS
PUC 610	-	494	Morro dos Conventos/RS
TR 1	230	557	Praia de Jaguaruna/SC
TR 2	160	508	Praia de Jaguaruna/SC
TR 4	225	483	Praia de Jaguaruna/SC
TR 5	310	485	Praia de Jaguaruna/SC
TR 6	280	526	Praia de Jaguaruna/SC
TR 7	300	507	Praia de Jaguaruna/SC
TR 8	250	515	Praia de Jaguaruna/SC
TR 201	-	471	Morro dos Conventos/SC
TR 544	-	500	Praia da Gaivota/RS
TR 554	353	451	Praia da Cal, Torres/RS
TR 567	-	519	Praia de Jaguaruna/SC
TR 632	-	449	Praia da Cal, Torres/RS
C. minutus - Sympatry			
ID	Body mass (g)	Skull lenght (mm)	Locality
TR 40	250	480	Praia do Barco/RS
TR 41	250	374	Praia do Barco/RS
TR 42	90	505	Praia do Barco/RS
TR 43	110	392	Praia do Barco/RS
TR 46	200	481	Praia do Barco/RS
TR 52	250	489	Capão Novo/RS
TR 55	240	460	Capão Novo/RS
TR 56	220	564	Capão Novo/RS
TR 1841	125	464	Praia do Barco/RS
TR 1843	170	507	Praia do Barco/RS
TR 1844	265	515	Praia do Barco/RS
TR 1846	275	443	Praia do Barco/RS
TR 1847	150	432	Praia do Barco/RS
TR 1848	165	443	Praia do Barco/RS
TR 1850	125	491	Praia do Barco/RS
TR 1851	115	468	Praia do Barco/RS
TR 1855	150	461	Praia do Barco/RS
TR 1856	130	521	Praia do Barco/RS
TR 1857	270	492	Praia do Barco/RS
TR 1858	195	453	Praia do Barco/RS
TR 1860	185	446	Praia do Barco/RS

TR 1862	180	486	Praia do Barco/RS

SUPPLEMENTARY INFORMATION 7 – Description of landmark positions.

L1: Anterior tip of suture between premaxillas; L2,3: lateral extremity of incisor alveolus; L4,5: lateral edge of incisive foramen in the suture between premaxilla and maxilla; L6,7: anteriormost point of the root of zygomatic arch; L8,9: anteriormost point of premolar alveolus; L10,11: anteriormost point of the orbit in inferior zygomatic root; L12,13: posterior extremity of third molar alveolus; L14: posterior extremity of suture between palatines; L15,16: anteriormost point of intersection between jugal and squamosal; L17,18: posteriormost point of pterygoid; L19,20: anteirior extremity of tympanic bulla; L21,22: anterior tip of external auditory meatus; L23,24: posterior extremity of mastoid apophysis; L25,26: posterior extremity of paraoccipital apophysis; L27: anteriormost point of foramen magnum; L28: posteriormost point of foramen magnum at midsagittal plane; L29,30: posterior extremity of occipital condyle in foramem magnum.
